# Safety and efficacy of biological agents in the treatment of Systemic Lupus Erythematosus (SLE)

**DOI:** 10.1186/s41927-023-00358-3

**Published:** 2023-10-09

**Authors:** Justin Chan, Giles D. Walters, Prianka Puri, Simon H. Jiang

**Affiliations:** 1 John Curtin School of Medical Research, Australian National University, Canberra, Australian Capital Territory Australia; 2 Department of Renal Medicine, Canberra Hospital, Canberra, Australian Capital Territory Australia; 3 Department of Nephrology, Royal Brisbane and Woman’s Hospital Health Service District, Herston, QLD Australia

**Keywords:** Systemic lupus erythematosus, Renal lupus, Biologics

## Abstract

**Background:**

To determine the safety and efficacy of biological agents used in the treatment of systemic lupus erythematosus (SLE) in adults.

**Methods:**

Systematic review and meta-analysis following PRISMA guidelines.

**Data sources:**

MEDLINE (through Pubmed), EMBASE, Cochrane library, Clinicaltrials.gov, Australianclinicaltrials.gov.au, ANZCTR.org.au and WHO International Clinical Trials Registry Platform for studies published from 20 May 2021 and 15 years prior. A grey literature search was performed and completed on 31 May 2021.

**Study criteria:**

Phase II, III or quasi randomised controlled trials, studies with only cerebral or cutaneous lupus were excluded. Data extraction: Two authors independently screened studies for eligibility, extracted, reviewed data for accuracy, and used the Cochrane tool to assess risk of bias.

**Results:**

Forty-four studies were identified, consisting of 15 groups of drugs and 25 different biological agents, totalling 16,889 patients. The main outcomes assessed included Systemic Lupus Erythematosus Responder Index (SRI), BILAG-Based Composite Lupus Assessment (BICLA) and combined combined/partial renal remission (CRR/PRR).

Four groups of biologics were found to improve outcomes. Anti-interferons: Anifrolumab increased BICLA response and SRI 5 to 8, decreased prednisone dosages, with increased herpes zoster infections, but fewer serious adverse events. Sifalimumab improved SRI but also increased herpes zoster infections. Anti BAFF/BLyS and/or APRIL: Belimumab consistently improved SRI 4, decreased prednisone dosages, increased combined CRR/PRR, and had no adverse safety outcomes. Tabalumab increased SRI 5 at 52 weeks with no steroid sparing effect but was associated with increased infusion related adverse events. Telitacicept improved SRI 4 at 52 weeks, with no increased adverse events, though data was rather sparse. Anti CD-20 monoclonal antibody, Obinutuzumab increased combined CRR/PRR at 1 and 2 years. Anti IL12/23 monoclonal antibody, Ustekinumab, increased SRI 4 to 6, but not BICLA at 24 weeks, with no concerning safety outcomes.

**Conclusion:**

Multiple biologic agents are shown in high quality studies to have a significant therapeutic impact on outcomes in SLE.

**Supplementary Information:**

The online version contains supplementary material available at 10.1186/s41927-023-00358-3.

## Background

Systemic Lupus Erythematosus (SLE) is an autoimmune disease of unknown aetiology with multiple manifestations including musculoskeletal, renal, haematological, serosal, and neuropsychiatric involvement. Treatment for SLE to date is centred on immunosuppression and anti-inflammatory therapy, depending on the degree of end organ involvement. Pregnant and non-pregnant lupus patients benefit from the use of hydroxychloroquine (HCQ), with reductions in lupus flares, end organ damage, loss of bone mass, thrombosis, cumulative steroid usage and increased long term survival [1]. Non-steroidal anti-inflammatory drugs (NSAIDs) may be used to manage milder manifestations such as musculoskeletal or mucocutaneous manifestations. Chronic glucocorticoid (GC) therapy is associated with cumulative dose toxicity. However, given its efficacy it is often used in lower doses as a component of maintenance therapy, or in higher doses for the treatment of disease flares depending on the severity of end organ involvement. Other immunosuppressants used include mycophenolate mofetil (MMF), cyclophosphamide (CYC) and calcineurin inhibitors such as tacrolimus (TAC) and azathioprine (AZA). “Standard of care” therapy is typically defined in clinical trials to include these agents.

Multiple biological agents have recently emerged as potential novel treatments for SLE. In this review we aim to summarise the available data from randomised controlled trials for the efficacy of biologics in SLE, and to highlight potential therapies which require further data.

## Methods

All phase II, and III clinical trials or randomised control trials or quasi randomised controlled trial enrolling adult patients with SLE according to standard criteria, examining biologic agent/s compared to placebo, other immunosuppressive drug/s or standard of care were examined.

Outcome measures included change in validated disease activity indices such as SLEDAI, SELENA-SLEDAI, SLEDAI-2 K, BILAG, BILAG-2004, SLICC/ACR score. Adverse events and death were also recorded.

Search methods are documented in the online supplement. Two authors independently examined all studies and extracted data. Dichotomous outcome results were expressed as risk ratios (RR) with 95% confidence intervals (CI), with data pooled using random effects models. Data with continuous outcomes were not measured in this review. The Cochrane risk of bias tool was used by both authors to independently assess the quality of included studies.

## Results

**Fig. 1:**
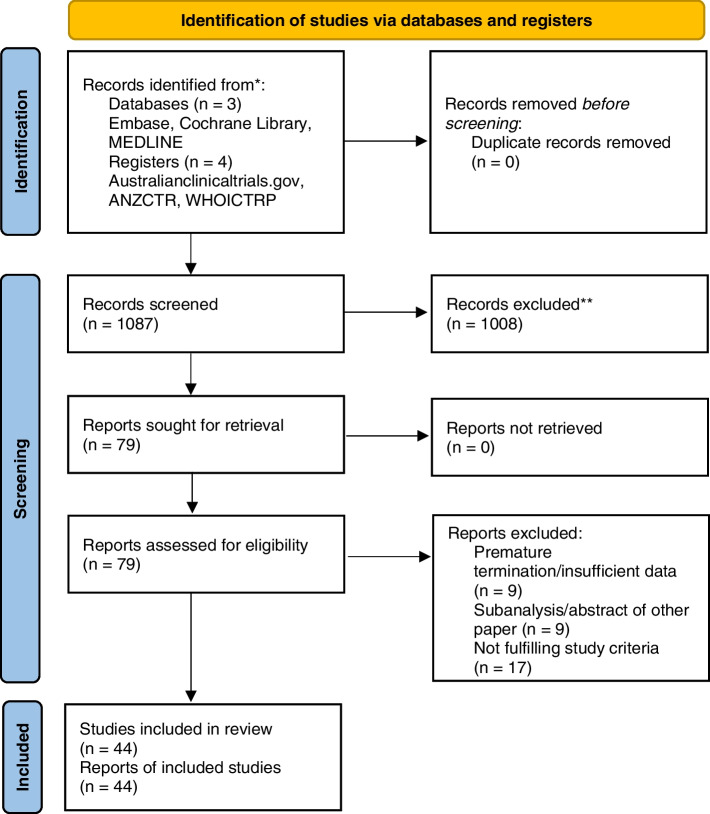
PRISMA flow

One thousand eighty-seven studies were identified. Seventy-nine studies were further assessed. Forty-four studies were included with 16,889 patients, 15 distinct drug groups and 25 biological agents. Characteristics of the studies including patient characteristics and study protocols are summarised in the supplementary information. PRISMA flow diagram is shown below (Fig. 1), and the PRISMA checklist is included in the supplementary information.

### CD80/86 inhibition

CD80/86 is expressed by antigen presenting cells such as plasmacytoid dendritic cells and B cells. CD80/86 ligate CD28, a co-stimulatory receptor expressed on T cells. CD28 stimulation in conjunction with T cell receptor engagement prolongs and increases T cell differentiation and production of IL2, with subsequent B cell proliferation and differentiation into antibody producing plasma cells.

### Abatacept

Abatacept is a fusion protein composed of a CTLA-4 molecule linked to the Fc portion of IgG1. This selectively and competitively antagonises CD80 and CD86 receptors on an antigen presenting cell, limiting CD28 mediated T cell activation.

Four studies [2–5] included 1017 patients. Three of the studies recruited patients with lupus nephritis whereas Merrill 2010 [2] excluded patients with renal involvement.

**Fig. 2:**
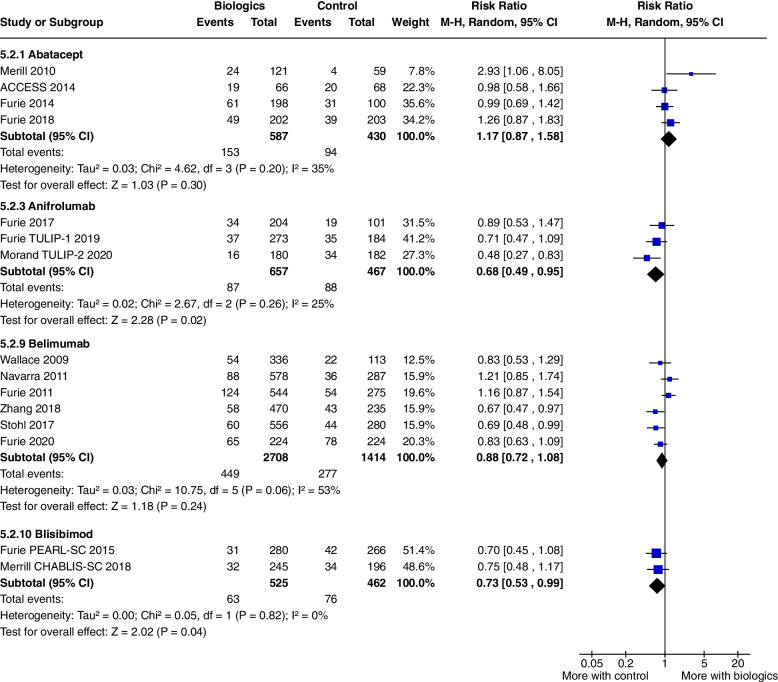
Serious adverse events

No outcomes achieved significance. Serious adverse events were significantly raised only in Merrill 2010 (RR 2.93, CI 1.06 to 8.05, *P* = 0.04) but not in the pooled data of all the Abatacept studies (RR 1.17, CI 0.87 to 1.58, *P* = 0.30) (Fig. 2).

### Anti-interferon monoclonal antibody

T1 IFN is considered the canonical SLE cytokine impairing immune tolerance through multiple mechanisms. Three anti-interferon monoclonal antibodies have been assessed in this review. Anifrolumab which binds to both IFN-α/β receptors, Rontalizumab and Sifalimumab which selectively bind to IFN-α receptors.

### Anifrolumab

Anifrolumab is a fully human, IgG1k monoclonal antibody that binds to IFN-α/β receptor and prevents signalling by all types of I IFNs.

Three studies addressed the use of Anifrolumab in SLE: Furie 2017 [6], Furie 2019 [7] and Morand 2020 [8] and included 1124 patients. The main outcomes studied were SRI and BICLA response.

**Fig. 3:**
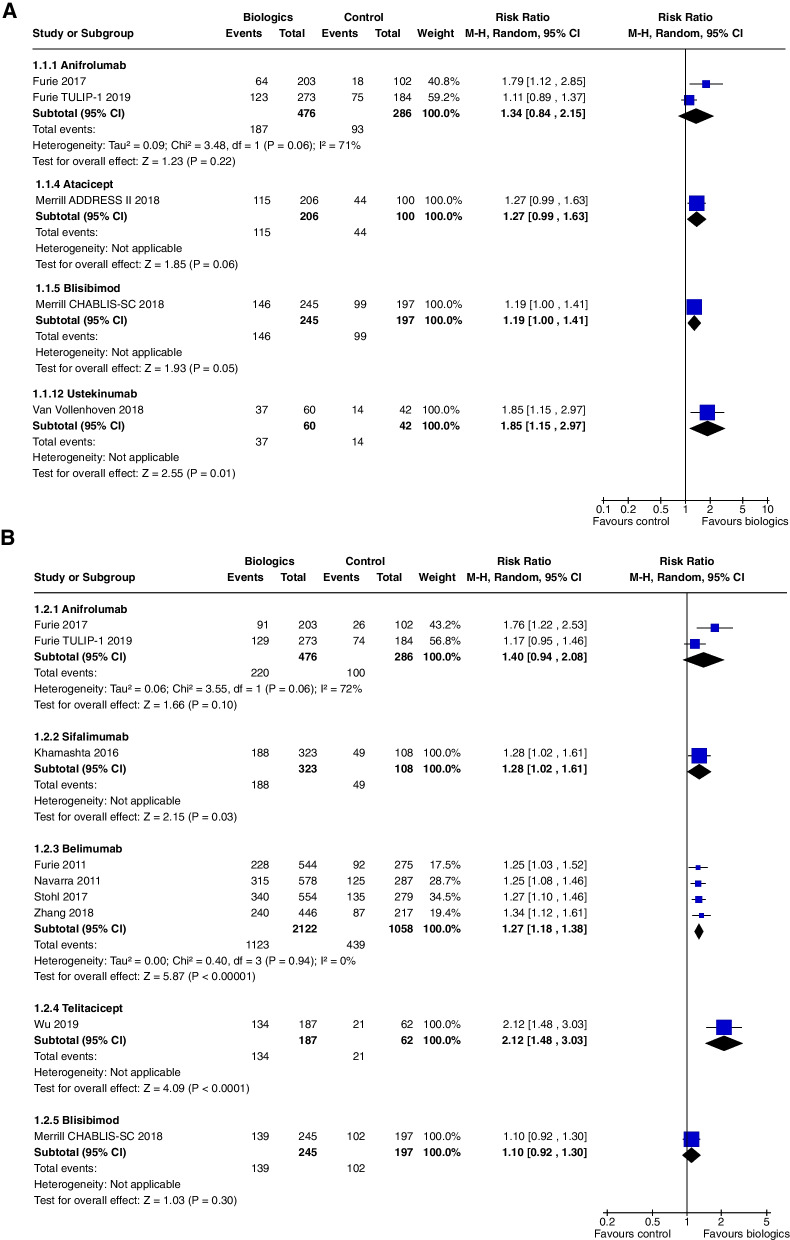
**A** SRI 4 at 24 weeks, **B** SRI 4 at 52 weeks

SRI 4 at 24 weeks did not achieve statistical significance (RR 1.34, CI 0.84 to 2.15, *P* = 0.22, 2 studies [6, 7]), though results from Furie 2017 alone were significant (RR 1.79, CI 1.12 to 2.85, *P* = 0.01) (Fig. 3a).

SRI 4 at 52 weeks did not achieve statistical significance (RR 1.40, CI 0.94 to 2.08, *P* = 0.10, 2 studies [6, 7]), though results from Furie 2017 alone were significant (RR 1.76, CI 1.22 to 2.53, *P* = 0.002) (Fig. 3b).

**Fig. 4:**
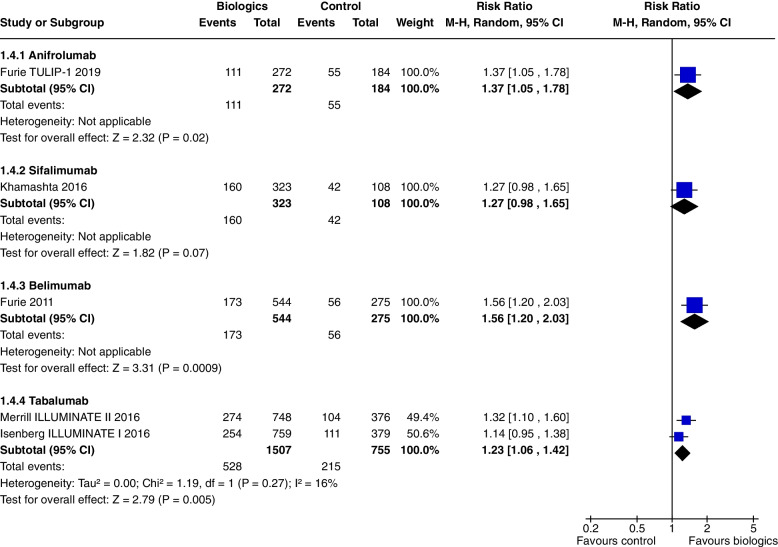
SRI 5 at 52 weeks

**Fig. 5:**
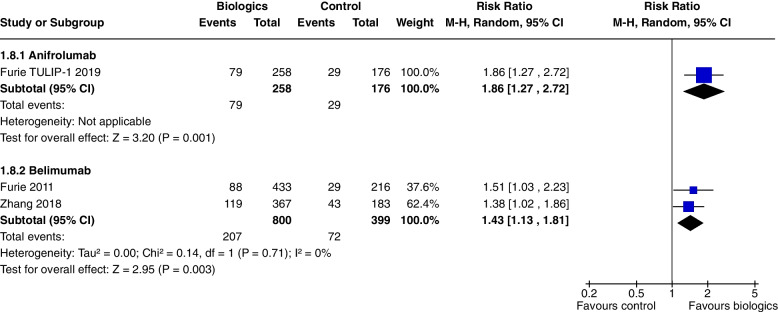
SRI 7 at 52 weeks

**Fig. 6:**
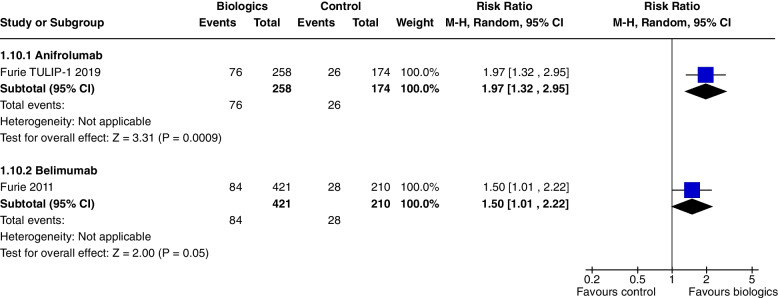
SRI 8 at 52 weeks

**Fig. 7:**
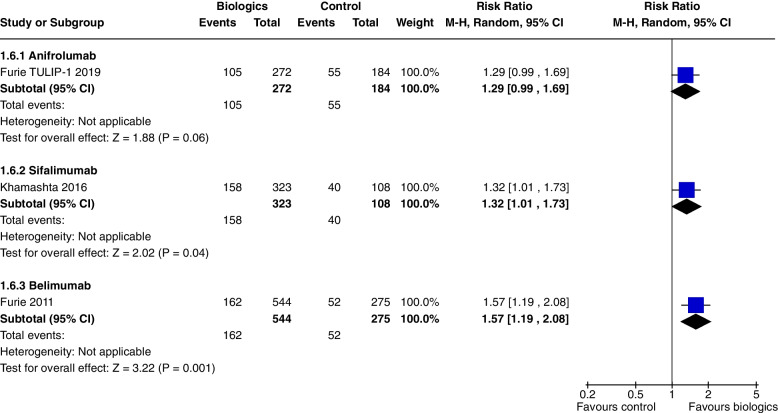
SRI 6 at 52 weeks

In a single study [7] at 52 weeks, Anifrolumab significantly increased SRI 5 (RR 1.37, CI 1.05 to 1.78, *P* = 0.02) (Fig. 4), SRI 7 (RR 1.86, CI 1.27 to 2.72, *P* = 0.001) (Fig. 5), and SRI 8 (RR 1.97, CI 1.32 to 2.95, *P* = 0.0009) (Fig. 6), but not SRI 6 (RR 1.29, CI 0.99 to 1.69, *P* = 0.06), though the results trended towards significance (Fig. 7).

**Fig. 8:**
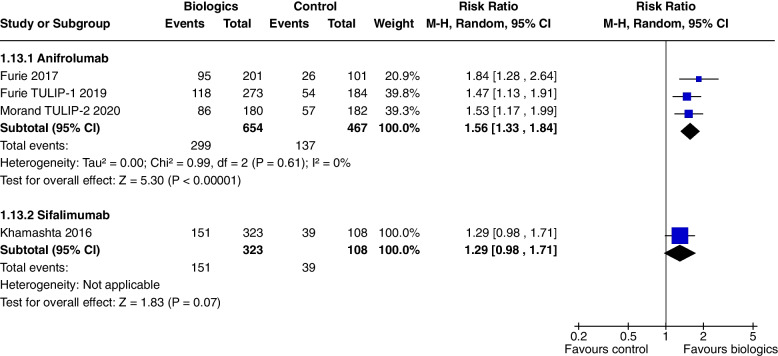
BICLA at 52 weeks

Anifrolumab significantly increased BICLA response at 52 weeks in all 3 studies (RR 1.56, CI 1.33 to 1.84, *P* < 0000.1) (Fig. 8).

**Fig. 9:**
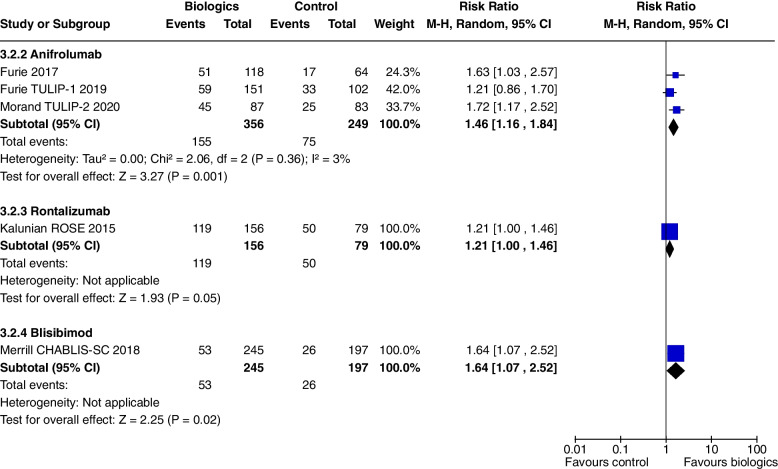
Change in prednisone dosages to ≤ 10 mg/day

Prednisone dose reduction to < 10 mg/day was increased with Anifrolumab treatment (RR 1.46, CI 1.16 to 1.84, *P* = 0.001, 3 studies) (Fig. 9).

**Fig. 10:**
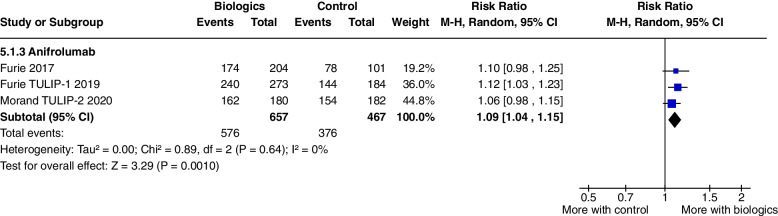
Adverse events

Adverse events were increased with Anifrolumab treatment, (RR 1.09, CI 1.04 to 1.15, *P* = 0.001, 3 studies) (Fig. 10), with a higher incidence of herpes zoster infections, but there were significantly fewer serious adverse events, (RR 0.68, CI 0.49 to 0.95, *P* = 0.02, 3 studies) compared to controls (Fig. 2). The other safety outcomes did not reach statistical significance.

### Rontalizumab

Rontalizumab is a human anti-IFN-α monoclonal antibody that binds to all 12 IFN-α subtypes preventing signalling through the type I IFN receptor.

One study [9] including 238 patients addressed the use of Rontalizumab in SLE: Kalunian 2016 Patients with lupus nephritis were excluded. At 24 weeks, Rontalizumab did not improve SRI 4 (RR 1.11, CI 0.83 to 1.48, *P* = 0.47) (Fig. 3a), though there were steroid sparing benefits with an increased number of patients tapering their steroids to a prednisone equivalent of ≤ 10 mg/day (RR 1.21, CI 1.0 to 1.46, *P* = 0.05) (Fig. 9). There were no significant differences in safety outcomes.

### Sifalimumab

Sifalimumab is a fully human, immunoglobulin G1 κ monoclonal antibody that binds to and neutralises the majority of IFN-α subtypes.

**Fig. 11:**
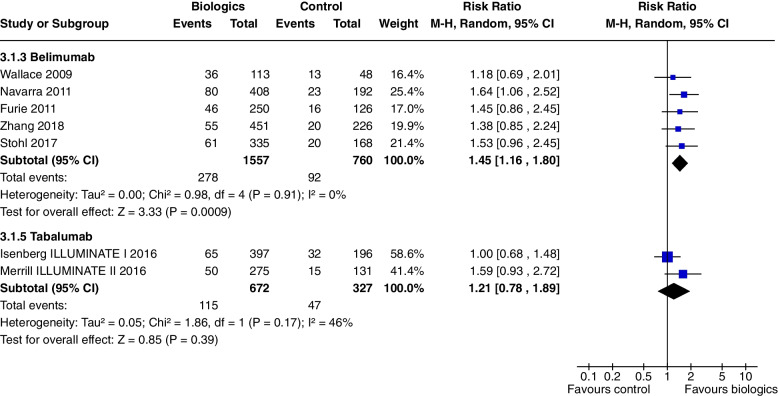
Change in prednisone dosages to ≤ 7.5 mg and > 25% reduction from baseline dosage

In a single study [10], at 52 weeks, Sifalimumab improved SRI 4, (RR 1.28, CI 1.02 to 1.61, *P* = 0.03) (Fig. 3b) and SRI 6 (RR 1.32, CI 1.01 to 1.73, *P* = 0.04) (Fig. 7). SRI 5 at 52 weeks (RR1.27, CI 0.98 to 1.65, *P* = 0.07) (Fig. 4) and BICLA at 52 weeks (RR 1.29, CI 0.98 to 1.71, *P* = 0.07) (Fig. 8) trended towards but did not achieve significance. There were no steroid sparing benefits, with no difference in the reduction in prednisone < 7.5 mg/day with 25% reduction from baseline dosage (RR 1.21, CI 0.41 to 3.54, *P* = 0.73) (Fig. 11). Adverse events were not increased with the use of Sifalimumab, though there were higher rates of herpes zoster compared to placebo (5.9% vs 0.9%).

### Anti BAFF/BLyS and APRIL monoclonal antibody

BAFF and APRIL are cytokines from the TNF family, secreted by most myeloid and lymphoid cells, and bind to TACI, BCMA and BAFF receptors. Ligation of BAFF receptors promote B cell survival, immunoglobulin class switching and secretion. BAFF binds to all 3 receptors, whereas APRIL only binds to TACI and BCMA. Blisibimod and Tabalumab inhibit soluble and membrane bound BAFF and Belimumab binds to soluble human BAFF. Atacicept and Telitacicept block both BlyS and APRIL.

### Belimumab

Belimumab is a human IgG1 monoclonal antibody that binds soluble human BlyS. It is currently only indicated for use in SLE not responding to standard of care therapy.

Seven studies [11–17] including 4022 patients. Furie 2020 [16] and Atisha Fregoso 2021 [17] included patients with lupus nephritis.

At 52 weeks, Belimumab use significantly increased SRI 4 (RR 1.27, CI 1.18 to 1.38, *P* < 0.0001, 4 studies) (Fig. 3b). In a single study at 52 weeks, improvements were demonstrated in SRI 5 (RR 1.56, CI 1.20 to 2.03, *P* = 0.0009) (Fig. 4), SRI 6 (RR 1.57, CI 1.19 to 2.08, *P* = 0.001) (Fig. 7) and SRI 8 (RR 1.50, CI 1.01 to 2.22, *P* = 0.05) (Fig. 6). In two studies, SRI 7 at 52 weeks significantly increased (RR 1.43, CI 1.13 to 1.81, *P* = 0.003) (Fig. 5).

**Fig. 12:**
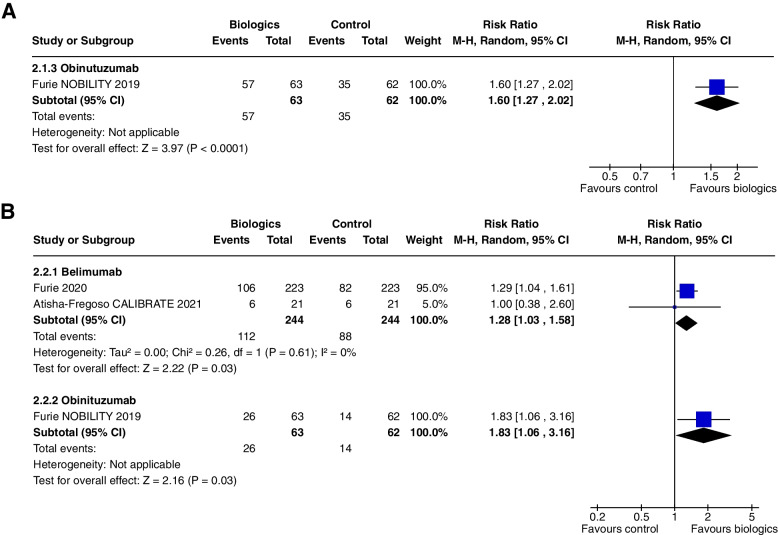
**A** Combined complete and partial renal remission at 1 year, **B** combined complete and partial renal remission at 2 years

Belimumab did not alter CRR/PRR at 1 year (RR 1.28, CI 0.67 to 2.45, *P* = 0.45, 1 study) but showed a significant effect at 2 years (RR 1.29, CI 1.04 to 1.61, *P* = 0.03, 2 studies) (Fig. 12b).

Belimumab significantly increased the number of patients able reduce prednisone dosages to ≤ 7.5 mg/day (RR 1.45, CI 1.16 to 1.80, *P* = 0.0009, 5 studies (Fig. 11).

There were no significant difference in serious adverse events (RR 0.88, 0.72 to 1.08, *P* = 0.24, 6 studies) (Fig. 2) or in other reported safety outcomes.

### Blisibimod

Blisibimod is a selective inhibitor of soluble BAFF and membrane-bound BAFF, composed of a tetrameric BAFF binding domain fused to a human IgG1. Two studies [18, 19] included 988 patients. Patients with severe lupus nephritis were excluded.

Blisibimod increased SRI 4 at 24 weeks only in Merrill 2018 [19], (RR 1.19, CI 1.00 to 1.41, *P* = 0.05, 2 studies) (Fig. 3a), but not SRI 4 and 6 at 52 weeks or SRI 5 to 8 at 24 weeks. Blisibimod reduced prednisone dosage below 10 mg/day (RR 1.64, CI 1.07 to 2.52, *P* = 0.02, 1 study [19] (Fig. 9).

**Fig. 13:**
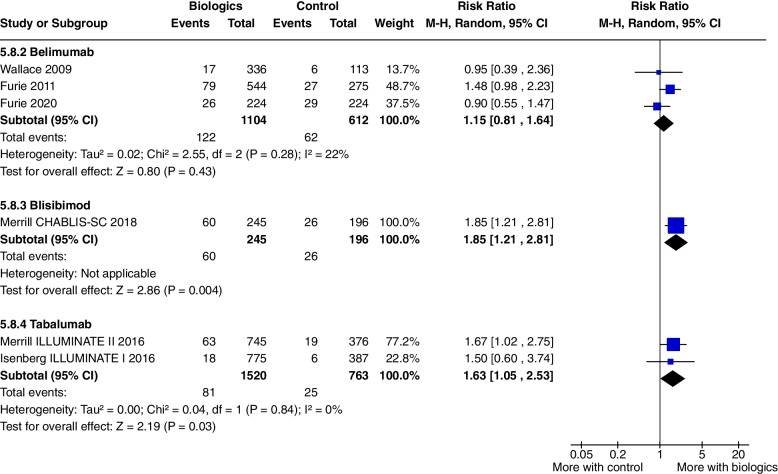
Infusion related adverse events

Infusion related adverse events were increased (RR 1.85, CI 1.21 to 2.81, *P* = 0.004, 1 study [19] (Fig. 13). There were no significant increase in other adverse events.

### Tabalumab

Tabalumab is a fully human IgG4 monoclonal antibody, that binds and neutralises both membrane and soluble BAFF. Two studies [20, 21] included 2262 patients. Patients with severe lupus nephritis were excluded.

Tabalumab significantly increased SRI 5 at 52 weeks (RR 1.23, CI 1.06 to 1.42, *P* = 0.005, 2 studies) (Fig. 4). Tabalumab did not significantly decrease prednisone doses (RR 1.21, CI 0.78 to 1.89, *P* = 0.39, 2 studies).

Infusion related adverse events were significantly higher with Tabalumab, (RR 1.63, CI 1.05 to 2.53, *P* = 0.03, 2 studies) (Fig. 13). Tabalumab did not increase withdrawals from the study, serious infections or death.

### Atacicept

Atacicept is a recombinant fusion protein comprising the extracellular domain of the TACI receptor joined to a human IgG1 Fc domain that blocks B-cell activating factor BlyS and APRIL.

Two studies [22, 23] included 767 patients. Patients with lupus nephritis were excluded.

In one study [23], Atacicept did not increase SRI 4 (RR 1.27, CI 0.99 to 1.63, *P* = 0.06) (Fig. 3a), SRI 6 (RR 1.13, CI 0.79 to 1.62, *P* = 0.49) and BICLA (RR 1.13, CI 0.87 to 1.47, *P* = 0.36) at 24 weeks.

There were no steroid sparing benefits or significant differences in the safety outcomes.

### Telitacicept

Telitacicept is a fusion protein comprising a recombinant TACI receptor fused to the Fc domain of human IgG, which binds to and neutralises the BLyS and APRIL, suppressing development and maturation of plasma cells and mature B cells.

One study [24] included 202 patients. Patients with severe lupus nephritis were excluded.

SRI 4 at 52 weeks was significantly increased with Telitacicept (RR 2.12, CI 1.48 to 3.03, *P* < 0.00001) (Fig. 3b).

There were no significant differences in reported safety outcomes of adverse events, serious adverse events and death.

### Anti-CD20 monoclonal antibody

Three anti CD20 monoclonal antibodies are examined in this review, Rituximab (murine-human chimeric), Ocrelizumab and Obinutuzumab (humanised).

### Obinutuzumab

Obinutuzumab is a recombinant type II anti-CD20 and IgG1 Fc-optimised humanised monoclonal antibody, which has improved mAb-FcγRIIIA interaction and direct and immune effector cell-mediated cytotoxicity compared to Rituximab.

One study [25] included 125 patients. Patients with lupus nephritis ISPN/RPS 2003 class III/IV were included in the study.

Combined CRR/PRR at 1 year was increased (RR 1.60, CI 1.27 to 2.02, *P* < 0.0001) (Fig. 12a) and 2 years (RR 1.83, CI 1.06 to 3.16, *P* = 0.03) (Fig. 12b).

**Fig. 14:**
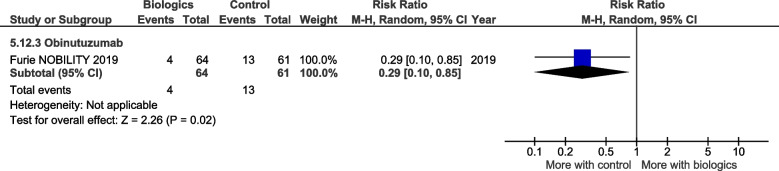
Grade 3 or higher related infectious events

There were fewer grade 3 or higher related infectious events with Obinutuzumab (RR 0.29, CI 0.10 to 0.85, *P* = 0.02) (Fig. 14), but no significant differences in the other safety outcomes.

### Ocrelizumab

Ocrelizumab is a humanised monoclonal antibody against CD20 and may have greater antibody dependent cellular toxicity and less complement dependent cytotoxicty compared to Rituximab which is a chimeric monoclonal antibody. One study [26] included 378 patients with lupus nephritis.

Combined CRR/PRR at 1 year was not increased (RR 1.22, CI 0.97 to 1.55, *P* = 0.09).

There were no significant differences in the pooled safety outcomes, but a higher rate of serious infections were seen in patients receiving MMF compared to ELNT induction.

### Rituximab

Rituximab is a type 1 chimeric anti-CD20 monoclonal antibody directed to the CD20 antigen on the surface of B lymphocytes, causing apoptosis, complement activation and cell mediated cytotoxicity.

Two studies [27, 28] included 401 patients. Rovin 2012 [28] only included patients with lupus nephritis class III/IV ± V. Rituximab did not increase CRR/PRR at 1 year, (RR 1.24, CI 0.90 to 1.71, *P* = 0.19, 1 study [28]). There was no reduction in the number of patients achieving prednisone < 10 mg/day (RR 0.81, CI 0.37 to 1.80, *P* = 0.60), 1 study [27].

### Ustekinumab

Ustekinumab is a fully humanised monoclonal antibody against the p40 subunit found on both IL-12 and IL-23. IL-12 has a key role in inducing Th cell differentiation to Th1 cells, and IL-23 in Th17 cell activation and subsequent IL-17 secretion.

One study [29] included 102 patients. Patients with lupus nephritis class III/IV were excluded.

**Fig. 15:**
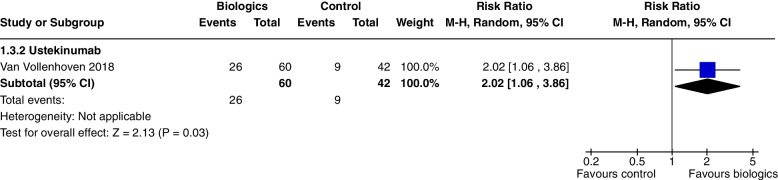
SRI 5 at 24 weeks

**Fig. 16:**
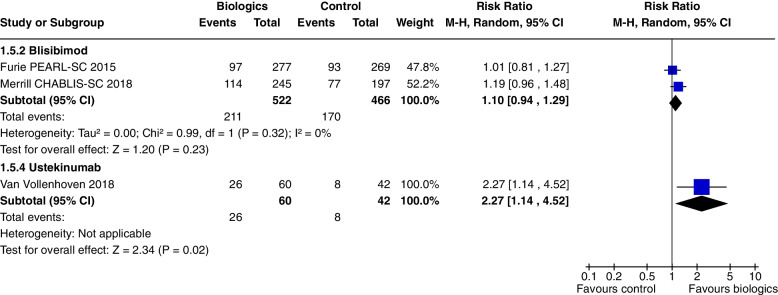
SRI 6 at 24 weeks

Ustekinumab increased SRI 4 at 24 weeks (RR 1.85, CI 1.15 to 2.97, *P* = 0.01) (Fig. 3a), SRI 5 at 24 weeks (RR 2.02, CI 1.06 to 3.86, *P* = 0.03) (Fig. 15) and SRI 6 at 24 weeks (RR 2.27, CI 1.14 to 4.52, *P* = 0.02) (Fig. 16), but not BICLA at 24 weeks (*P* = 0.86). Ustekinumab use did not increase any adverse events.

### Group of drugs without significant results

There were no significant outcomes in the disease activity indices, composite responder rates or adverse events in the following group of drugs; anti-dsDNA complexing Abetimus [30] selective JAK 1 and 2 inhibitors Baricitinib [31], BTK inhibitors Evobrutinib [32], Fenebrutinib [33], high affinity cereblon ligand CC-220/Iberdomide [34, 35], tolerogenic peptides Edratide [36], anti CD22 monoclonal antibody Epratuzumab [37–39], anti IL-6 antibody PF-04326921 [40] Vobarilizumab [41] anti IL-10 monoclonal antibody BT063 [42], P140 peptide Lupuzor [43] and recombinant soluble human FcyRIIb SM101 [44].

### Summary of findings

The main results of this review are presented in the summary of findings tables. Outcomes with significant results presented include composite outcomes, renal outcomes, glucocorticoid dose reduction, and adverse events. The complete GRADE tables are shown below (Refer Table 1: Composite outcomes, Table 2: Renal outcomes, Table 3: Glucocorticoid dose reduction, Table 4: Adverse events).

**Table 1:** Composite outcomes

Biologics compared to placebo for the treatment of Systemic Lupus Erythematosus measured by composite responder rates					
Patient or population: Systemic Lupus Erythematosus Setting: Inpatients then outpatients Intervention: Biologics Comparison: Standard of care, placebo					
Outcomes	№ of participants (studies) Follow-up	Certainty of the evidence (GRADE)	Relative effect (95% CI)	Anticipated absolute effects	
				Risk with Standard of care	Risk difference with Biologics
(SRI) 4 at 52 weeks—Anifrolumab	762 (2 RCTs)	⨁⨁⨁◯ Moderate^a^^,^^b^^,^^c^^,^^e^	RR 1.40 (0.94 to 2.08)	350 per 1,000	140 more per 1,000 (21 fewer to 378 more)
(SRI) 4 at 52 weeks—Sifalimumab	431 (1 RCT)	⨁⨁⨁◯ Moderate^f^	RR 1.28 (1.02 to 1.61)	454 per 1,000	127 more per 1,000 (9 more to 277 more)
(SRI) 4 at 52 weeks—Belimumab	3180 (4 RCTs)	⨁⨁⨁⨁ High^h^	RR 1.27 (1.18 to 1.38)	415 per 1,000	112 more per 1,000 (75 more to 158 more)
(SRI) 4 at 52 weeks—Telitacicept	249 (1 RCT)	⨁⨁◯◯ Low^d^^,^^e^^,^^f^^,^^g^	RR 2.12 (1.48 to 3.03)	339 per 1,000	379 more per 1,000 (163 more to 688 more)
BICLA response at 52 weeks—Anifrolumab	1121 (3 RCTs)	⨁⨁⨁◯ Moderate^a^^,^^b^^,^^c^^,^^i^	RR 1.56 (1.33 to 1.84)	293 per 1,000	164 more per 1,000 (97 more to 246 more)
BICLA response at 52 weeks—Sifalimumab	431 (1 RCT)	⨁⨁⨁◯ Moderate^e^^,^^f^	RR 1.29 (0.98 to 1.71)	361 per 1,000	105 more per 1,000 (7 fewer to 256 more)

GRADE Working Group grades of evidence

High certainty: we are very confident that the true effect lies close to that of the estimate of the effect

Moderate certainty: we are moderately confident in the effect estimate: the true effect is likely to be close to the estimate of the effect, but there is a possibility that it is substantially different

Low certainty: our confidence in the effect estimate is limited: the true effect may be substantially different from the estimate of the effect

Very low certainty: we have very little confidence in the effect estimate: the true effect is likely to be substantially different from the estimate of effect

*CI* Confidence interval, *RR* Risk ratio

The risk in the intervention group (and its 95% confidence interval) is based on the assumed risk in the comparison group and the relative effect of the intervention (and its 95% CI)

Explanations

^a^allocation concealment method not stated

^b^randomisation method not stated

^c^selective reporting, multiple analyses of data

^d^Wide CI

^e^single study

^f^Did not meet OIS criterion

^g^study protocols unavailable

^h^I2 = 0, significant P and consistently overlapping CI

^i^selective reporting, change in primary outcome resulting in significant outcome (Morand 2020)

**Table 2:** Renal outcomes

Biologics compared to placebo for the treatment of Systemic Lupus Erythematosus measured by renal outcomes					
Patient or population: Systemic Lupus Erythematosus Setting: Inpatients then outpatients Intervention: Biologics Comparison: Standard of care, placebo					
Outcomes	№ of participants (studies) Follow-up	Certainty of the evidence (GRADE)	Relative effect (95% CI)	Anticipated absolute effects	
				Risk with placebo	Risk difference with Renal outcomes
Partial and/or complete renal response by 1 year—Abatacept	377 (2 RCTs)	⨁⨁◯◯ Low^a^^,^^b^^,^^c^^,^^d^	RR 0.98 (0.78 to 1.23)	436 per 1,000	9 fewer per 1,000 (96 fewer to 100 more)
Partial and/or complete renal response by 1 year—Belimumab	43 (1 RCT)	⨁◯◯◯ Very low^c^^,^^d^^,^^e^	RR 1.28 (0.67 to 2.45)	409 per 1,000	115 more per 1,000 (135 fewer to 593 more)
Partial and/or complete renal response by 1 year—Obinutuzumab	125 (1 RCT)	⨁⨁⨁◯ Moderate^d^	RR 1.60 (1.27 to 2.02)	565 per 1,000	339 more per 1,000 (152 more to 576 more)
Partial and/or complete renal response by 1 year—Ocrelizumab	223 (1 RCT)	⨁⨁◯◯ Low^a^^,^^b^^,^^d^^,^^f^	RR 1.22 (0.97 to 1.55)	547 per 1,000	120 more per 1,000 (16 fewer to 301 more)
Partial and/or complete renal response by 1 year—Rituximab	144 (1 RCT)	⨁⨁⨁◯ Moderate^a^^,^^b^^,^^d^	RR 1.24 (0.90 to 1.71)	458 per 1,000	110 more per 1,000 (46 fewer to 325 more)
Partial and/or complete renal response by 2 years—Belimumab	488 (2 RCTs)	⨁◯◯◯ Very low^d^^,^^g^^,^^h^	RR 1.28 (1.03 to 1.58)	361 per 1,000	101 more per 1,000 (11 more to 209 more)
Partial and/or complete renal response by 2 years—Obinituzumab	125 (1 RCT)	⨁⨁⨁◯ Moderate^c^^,^^d^	RR 1.83 (1.06 to 3.16)	226 per 1,000	187 more per 1,000 (14 more to 488 more)

GRADE Working Group grades of evidence

High certainty: we are very confident that the true effect lies close to that of the estimate of the effect

Moderate certainty: we are moderately confident in the effect estimate: the true effect is likely to be close to the estimate of the effect, but there is a possibility that it is substantially different

Low certainty: our confidence in the effect estimate is limited: the true effect may be substantially different from the estimate of the effect

Very low certainty: we have very little confidence in the effect estimate: the true effect is likely to be substantially different from the estimate of effect

*CI* Confidence interval, *RR* Risk ratio

The risk in the intervention group (and its 95% confidence interval) is based on the assumed risk in the comparison group and the relative effect of the intervention (and its 95% CI)

Explanations

^a^randomisation method not specified

^b^allocation concealment method not specified

^c^Wide CI

^d^Not meeting OIS criteria

^e^not blinded, open label

^f^attrition bias, premature termination of study with incomplete reporting of primary endpoints

^g^not blinded, open label (Atisha Fregoso 2021)

^h^significant P value and 0% heterogeneity but CI from Atisha Fregoso wide and overlaps significant/non significance

**Table 3:** Glucocorticoid dose reduction

Biologics compared to placebo for the treatment of Systemic Lupus Erythematosus measured by glucocorticoid dose reduction					
Patient or population: Systemic Lupus Erythematosus 1. Setting: Inpatients then outpatients Intervention: Biologics Comparison: Standard of care, placebo					
Outcomes	№ of participants (studies) Follow-up	Certainty of the evidence (GRADE)	Relative effect (95% CI)	Anticipated absolute effects	
				Risk with placebo	Risk difference with Glucocorticoid dose
number of patients with prednisone equivalent ≤ 7.5 mg/day, with reduction ≥ 25% from baseline—Belimumab	2317 (5 RCTs)	⨁⨁⨁◯ Moderate^c^^,^^d^	RR 1.45 (1.16 to 1.80)	121 per 1,000	54 more per 1,000 (19 more to 97 more)
number of patients with prednisone equivalent ≤ 7.5 mg/day, with reduction ≥ 25% from baseline—Tabalumab	999 (2 RCTs)	⨁◯◯◯ Very low^a^^,^^b^^,^^d^^,^^e^	RR 1.21 (0.78 to 1.89)	144 per 1,000	30 more per 1,000 (32 fewer to 128 more)
number of patients with prednisone equivalent ≤ 10 mg/day—Anifrolumab	605 (3 RCTs)	⨁⨁◯◯ Low^a^^,^^d^^,^^e^^,^^f^	RR 1.46 (1.16 to 1.84)	301 per 1,000	139 more per 1,000 (48 more to 253 more)
number of patients with prednisone equivalent ≤ 10 mg/day—Rontalizumab	235 (1 RCT)	⨁⨁⨁◯ Moderate^a^^,^^c^^,^^d^	RR 1.21 (1.00 to 1.46)	633 per 1,000	133 more per 1,000 (0 fewer to 291 more)
number of patients with prednisone equivalent ≤ 10 mg/day—Blisibimod	442 (1 RCT)	⨁⨁⨁◯ Moderate^c^^,^^d^	RR 1.64 (1.07 to 2.52)	132 per 1,000	84 more per 1,000 (9 more to 201 more)

GRADE Working Group grades of evidence

High certainty: we are very confident that the true effect lies close to that of the estimate of the effect

Moderate certainty: we are moderately confident in the effect estimate: the true effect is likely to be close to the estimate of the effect, but there is a possibility that it is substantially different

Low certainty: our confidence in the effect estimate is limited: the true effect may be substantially different from the estimate of the effect

Very low certainty: we have very little confidence in the effect estimate: the true effect is likely to be substantially different from the estimate of effect

*CI* Confidence interval, *RR* Risk ratio

The risk in the intervention group (and its 95% confidence interval) is based on the assumed risk in the comparison group and the relative effect of the intervention (and its 95% CI)

Explanations

^a^allocation concealment method not stated

^b^Wide CI

^c^Single study

^d^Not meeting OIS criteria

^e^randomisation method not stated

^f^selective reporting bias, multiple analyses of data

**Table 4:** Adverse events

Biologics compared to placebo for the treatment of Systemic Lupus Erythematosus measured by adverse events					
Patient or population: Systemic Lupus Erythematosus Setting: Inpatients then outpatients Intervention: Biologics Comparison: Standard of care, placebo					
Outcomes	№ of participants (studies) Follow-up	Certainty of the evidence (GRADE)	Relative effect (95% CI)	Anticipated absolute effects	
				Risk with placebo	Risk difference with Adverse events
AEs—Anifrolumab	1124 (3 RCTs)	⨁⨁⨁◯ Moderate^a^	RR 1.09 (1.04 to 1.15)	805 per 1,000	72 more per 1,000 (32 more to 121 more)
AEs—CC-220	330 (2 RCTs)	⨁⨁◯◯ Low^a^^,^^b^	RR 1.23 (0.84 to 1.80)	363 per 1,000	83 more per 1,000 (58 fewer to 290 more)
Serious AEs—Abatacept	1017 (4 RCTs)	⨁⨁⨁◯ Moderate^a^	RR 1.17 (0.87 to 1.58)	219 per 1,000	37 more per 1,000 (28 fewer to 127 more)
Serious AEs—Anifrolumab	1124 (3 RCTs)	⨁⨁⨁◯ Moderate^a^	RR 0.68 (0.49 to 0.95)	188 per 1,000	60 fewer per 1,000 (96 fewer to 9 fewer)
Serious AEs—Belimumab	4122 (6 RCTs)	⨁⨁⨁◯ Moderate^e^	RR 0.88 (0.72 to 1.08)	196 per 1,000	24 fewer per 1,000 (55 fewer to 16 more)
Serious AEs—Blisibimod	987 (2 RCTs)	⨁⨁⨁◯ Moderate^a^	RR 0.73 (0.53 to 0.99)	165 per 1,000	44 fewer per 1,000 (77 fewer to 2 fewer)
Treatment related AEs—Belimumab	1989 (3 RCTs)	⨁⨁⨁◯ Moderate^a^	RR 1.12 (0.99 to 1.26)	334 per 1,000	40 more per 1,000 (3 fewer to 87 more)
Treatment related AEs—Blisibimod	987 (2 RCTs)	⨁⨁⨁⨁ High	RR 1.26 (0.89 to 1.78)	314 per 1,000	82 more per 1,000 (35 fewer to 245 more)
Treatment related AEs—CC-220	330 (2 RCTs)	⨁⨁⨁◯ Moderate^a^	RR 1.39 (1.02 to 1.90)	319 per 1,000	124 more per 1,000 (6 more to 287 more)
Infusion related AE—Belimumab	1716 (3 RCTs)	⨁⨁◯◯ Low^a^^,^^c^^,^^d^	RR 1.15 (0.81 to 1.64)	101 per 1,000	15 more per 1,000 (19 fewer to 65 more)
Infusion related AE—Blisibimod	441 (1 RCT)	⨁⨁⨁◯ Moderate^a^	RR 1.85 (1.21 to 2.81)	133 per 1,000	113 more per 1,000 (28 more to 240 more)
Infusion related AE—Tabalumab	2283 (2 RCTs)	⨁⨁⨁◯ Moderate^a^	RR 1.63 (1.05 to 2.53)	33 per 1,000	21 more per 1,000 (2 more to 50 more)
Infection related grade 3 or higher AE—Obinutuzumab	125 (1 RCT)	⨁⨁⨁◯ Moderate^a^	RR 0.29 (0.10 to 0.85)	213 per 1,000	151 fewer per 1,000 (192 fewer to 32 fewer)

GRADE Working Group grades of evidence

High certainty: we are very confident that the true effect lies close to that of the estimate of the effect

Moderate certainty: we are moderately confident in the effect estimate: the true effect is likely to be close to the estimate of the effect, but there is a possibility that it is substantially different

Low certainty: our confidence in the effect estimate is limited: the true effect may be substantially different from the estimate of the effect

Very low certainty: we have very little confidence in the effect estimate: the true effect is likely to be substantially different from the estimate of effect

*CI* Confidence interval, *RR* Risk ratio

The risk in the intervention group (and its 95% confidence interval) is based on the assumed risk in the comparison group and the relative effect of the intervention (and its 95% CI)

Explanations

^a^didn't meet OIS criteria

^b^high heterogeneity

^c^allocation concealment method not stated

^d^wide CI

^e^high heterogeneity and 2 studies suggesting reduction in events, 4 don't

### Risk of bias

**Fig. 17:**
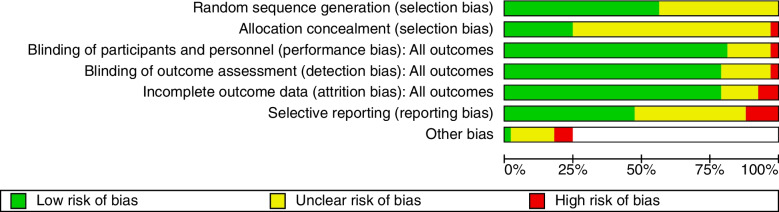
Risk of bias graph

**Fig. 18:**
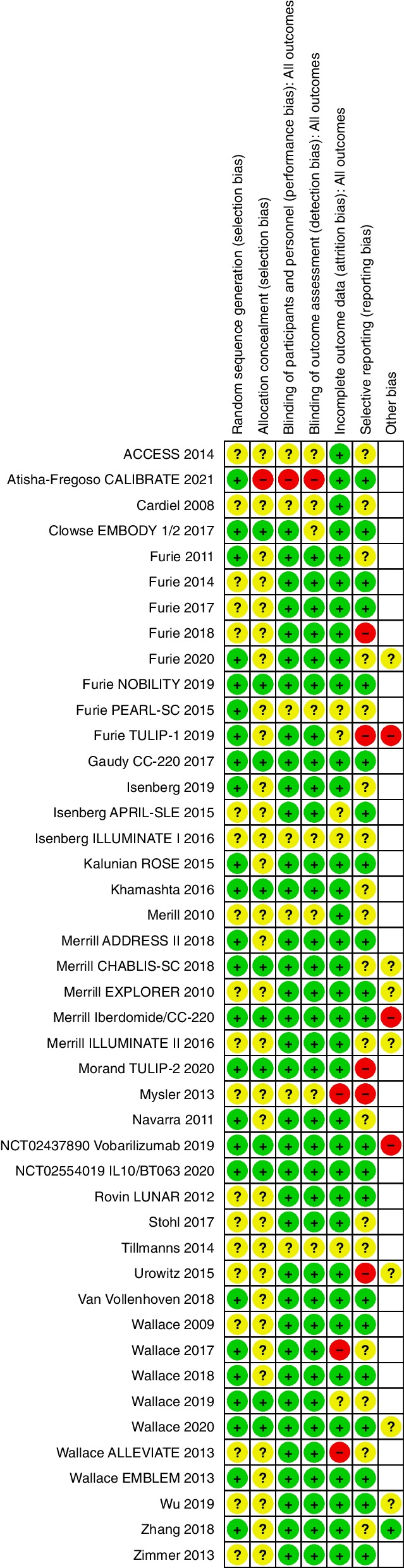
Risk of bias summary

Risk of bias graph (Fig. 17) and risk of bias summary (Fig. 18) is shown below.

## Discussion

Summary of main positive and negative outcomes of our study.

We have summarised the RCT data available on 25 biological agents from 15 different drug groups in the treatment of SLE. The majority of these drugs have limited data available and will require further trials to determine their efficacy in various patient groups.

Currently Belimumab is shown to have the most significant data suggesting that it is effective in SLE without a major adverse effect profile. There is high quality evidence showing Belimumab improves composite outcomes measured by SRI. The level of evidence for other biologics with significant outcomes range from low to moderate (Summary of findings: Composite outcomes). Other newer treatments have shown significant efficacy but in more specific outcomes and will need further trials to clearly delineate their strengths and weaknesses.

The main outcomes assessed in these studies were SRI, BICLA, and combined CRR/PRR. Of the 25 biologic agents, only anti-interferon, anti BAFF/BLyS and/or APRIL, anti IL12/23 and anti CD20 monoclonal antibodies were found to improve outcomes.

Anifrolumab increased BICLA response at 52 weeks, SRI 5 to 8 in a single study (Furie 2019), decreased prednisone dosages, with increased adverse events with herpes zoster infections, but with lesser serious adverse events. Sifalimumab also improved SRI but also increased herpes zoster infections. Among the anti BAFF/Blys and/or APRIL monoclonal antibodies, Belimumab consistently improved SRI 4, decreased prednisone dosages, increased combined CRR/PRR in a single study, and had no adverse safety outcomes. Tabalumab increased SRI 5 at 52 weeks with no steroid sparing effect but was associated with increased infusion related adverse events. Telitacicept also improved SRI 4 at 52 weeks, without data on its effect on steroid dosages. Of the three anti CD-20 monoclonal antibodies, only Obinutuzumab increased combined CRR/PRR at 1 and 2 years, with lower grade 3 or higher infectious events. The single anti IL12/23 monoclonal antibody, Ustekinumab, increased SRI 4 to 6, but not BICLA at 24 weeks, with no concerning safety outcomes.

Despite positive results in some of these biologics, several of their developments have since been terminated. There are no further trials planned for Tabalumab ( two phase III trials) by the parent pharmaceutical company as it was not felt to have reached significant efficacy compared to existing treatments, and Sifalimumab's development (one phase IIb trial) has been ceased in favour of Anifrolumab. Following the completion of this review, a phase III trial of Ustekinumab involving 516 patients showed no superiority compared to placebo when measuring SRI 4 as a primary endpoint [45].

Our review did not include non-biologics such as the calcineurin inhibitor Voclosporin which has shown benefit in proteinuria reduction in patients with lupus nephritis [46].

The other remaining drug classes and biological agents did not improve any of the outcomes assessed in the study and had no other notable safety outcomes.

### Difficulties with outcome measures

Prior to the introduction of SRI and BICLA, trials reported outcomes using individual BILAG, SLICC, SLEDAI based scores such as SLEDAI-2 K and SELENA-SLEDAI as their outcome measures. There were inconsistencies with how these scores were reported to denote significant results. Examples included BILAG as a numerical score determined by the study authors (and outcomes reporting changes in percentages, mean BILAG score differences compared to baseline) and differing organ domain severity scores (eg 1A and 2B, 1A and 1B, B only, C in all domains) and SLEDAI based metrics using varying decrease in points, expressed in means, medians or percentage of changes in baseline values.

SRI4 response is defined as SLEDAI improvement of 4 points or more, PGA not worsening by 0.3 points or more (10% or more), and BILAG having no new As and not having two or more new Bs. SRI 5, 6 and 7 correspond to an increase in improvement in SLEDAI points, without changes to the other criteria. BICLA response is defined as a reduction of all baseline BILAG-2004 A and B domain scores to B/C/D and C/D, no worsening in any organ system; no worsening of SLEDAI-2 K score from baseline, and no worsening ≥ 0.3 points (< 10% worsening) in Physician's Global Assessment, and no non-protocol treatment (new or increased immunosuppressives, antimalarials, corticosteroids or premature discontinuation of study treatment).

Comparing SRI and BICLA, SRI places more emphasis on SLEDAI improvement which does not evaluate the degree of individual component improvement, compared to the more comprehensive BILAG based BICLA, which does not evaluate for serological improvements. Ohmura 2021 [47] summarises the differences between the existing SLE activity indexes in clinical trials. Quality of life outcome measures also suffered from the aforementioned issues.

As SRI and BICLA incorporates a standardised change in BILAG, SELENA-SLEDAI/SLEDAI-2 K and PGA in their scoring, the authors of this study decided to omit data reporting other disease and quality of life outcomes outside of SRI and BICLA. This is mainly to maximise data that can be appropriately compared across studies, the main utility of a systematic review such as this.

Studies of lupus nephritis also did not use standardised definitions of complete or partial renal remissions (Table 5: Renal outcomes). Neither did they provide adequate reporting on other renal outcomes time to ESRD, or changes in serum creatinine/eGFR.

**Table 5:** Renal outcomes

Study	Complete renal remission	Partial renal remission
Abatacept		
ACCESS 2014 [4]	UPCR < 0.5 based on a 24-h urine collection, Serum creatinine ≤ 1.2 mg/dl or ≤ 125% of baseline Adherence to the prednisone taper to 10 mg/day by week12	UPCR 50% improvement from baseline Serum creatinine ≤ 1.2 mg/dl or ≤ 125% of baseline Adherence to the prednisone taper to 10 mg/day by week12
Furie 2014 [3]	EGFR 90% of screening level if normal at screening visit or eGFR 90% of 6-month, pre-flare value if abnormal at screening, UPCR 0.26 gm/gm (30 mg/mmole) Inactive urinary sediment (RBCs and WBCs per hpf within normal limits, no RBC or WBC casts All complete response criteria had to be met once again, 4 weeks after they were initially achieved	Inactive urinary sediment regardless of the screening value UPCR 50% improvement from screening value eGFR ≥ 90% of screening value if eGFR 60–89 ≥ 50% improvement in eGFR if screening eGFR was between 15–59, or eGFR ≥ 90% of the screening or 6 month pre-flare value
Furie 2018 [5]	Maintenance of GFR UPCR ≤ 0.5 Absence of urinary cellular casts Pednisone ≤ 10 mg/day	None
Belimumab		
Furie 2020 [16]	UPCR of < 0.5 eGFR that was no worse than 10% below the preflare value or ≥ 90 No rescue therapy	≥ 50% decrease in the uPCR and either uPCR < 1.0, if the baseline ratio was ≤ 3.0 or < 3.0, if the baseline ratio was > 3.0
Atisha-Fregoso 2021 [17]	UPCR of < 0.5 based on a 24-h urine sample collection eGFR of ≥ 120 or if the value was < 120, then > 80% of the eGFR recorded at the time of study entry Adherence to the prednisone dosing provisions. (prednisone 40 mg/day with taper to 10 mg/day by week 12, and ≤ 10 mg/day through week 96.)	eGFR no more than 10% below the baseline value or within normal range
Ocrelizumab		
Mysler 2013 [26]	Serum creatinine ≤ 25% increase from baseline UPCR < 0.5	Serum creatinine ≤ 25% above baseline 50% improvement in UPCR, if baseline ratio > 3.0, then UPCR < 3.0
Obinutuzumab		
Furie 2019 [7]	Maintenance of eGFR, UPCR) ≤ 0.5 Absence of urinary cellular casts Prednisone ≤ 10 mg/day	Serum creatinine ≤ 15% above baseline value No urinary red cell casts and either RBCs/HPF ≤ 50% above baseline or < 10 RBCs/HPF 50% improvement in UPCR, with one of following conditions met: If baseline UPCR is ≤ 3.0, then a UPCR of < 1.0 If baseline UPCR > 3.0, then a UPCR of < 3.0
Rituximab		
Rovin 2012 [28]	Normal creatinine level if it was abnormal at baseline or a creatinine level of ≤ 115% of baseline if it was normal at baseline Inactive urinary sediment (< 5 RBCs/hpf and absence of RBC casts); and UPCR ratio < 0.5	Creatinine level ≤ 115% of baseline RBCs/hpf ≤ 50% above baseline and no RBC casts At least a 50% decrease in the UPC ratio to < 1.0 (if the baseline UPC ratio was ≤ 3.0) or to ≤ 3.0 (if the baseline UPC ratio was > 3.0)

### Comparison with other systematic reviews

Four other reviews examined the use of biologic agents in the treatment of SLE. A meta-analysis by Oon 2018 [48] that Belimumab, Tabalumab and Epratuzumab had steroid sparing effects, which differed from our finding of only Belimumab had a significant steroid sparing effect. We did not include the data of steroid doses in the Epratuzumab studies of Wallace (EMBLEM) 2013 and Clowse 2017 as they were reported as mean ± SD and Wallace 2013 (ALLEVIATE) which reported them as medians without sufficient IQR data. Borba 2014 [49] which assessed 7 biologic agents similarly concluded that Belimumab improved disease response in the outcomes assessed compared to placebo. Singh 2021 [50] assessed 6 RCTs of Belimumab and concluded that Belimumab was effective in increasing SELENA-SLEDAI (≥ 4 point improvement) and reduction in glucocorticoid dosages. Sciascia 2017 [51] assessed the efficacy of Belimumab in renal outcomes and reported a decrease in proteinuria in patients treated with Belimumab but were unable to arrive to any conclusions for other parameters of renal response due to differing criteria across the studies. We did not include data describing renal outcomes such as number of and time to renal flares, and proteinuria due to the heterogeneous methods of reporting them across the studies, limiting their applicability in a systematic review.

## Conclusions

### Recommendations for patient treatments

Based on current data, Anifrolumab, Sifalimumab, Belimumab, Tabalumab, Telitacicept, are effective treatments in the treatment of SLE without lupus nephritis. Anifrolumab and Belimumab are useful in decreasing the steroid burden in these patients when compared to other biologics. In patients with lupus nephritis, Belimumab and Obinutuzumab are effective treatments. There is insufficient data to recommend for or against the use of biologics in CNS lupus due to their exclusion from trials. Patients treated with Anifrolumab or Sifalimumab should consider herpes zoster vaccination prior to commencing treatment.

### Recommendations for further research

Our review has revealed and summarised a wealth of studies in the treatment of SLE with biological agents and demonstrated the limited availability of data in many of these agents and the need for further studies to elucidate the efficacy of each agent in SLE treatment.

Comparison between agents will need to emerge as a research question in the near future. Other potential areas to consider will be the combination of treatments from different drug groups to improve the overall efficacy of disease control over time.

Studies involving biologics in SLE have heterogeneous endpoints and duration. The majority of the studies selectively excluded renal lupus involvement, though the criteria for exclusion varied widely, from active urinary sediment and mildly decreased eGFR to rapidly progressing glomerulonephritis. As lupus nephritis remains a leading cause of morbidity and mortality in SLE, a larger number of trials with a standardised definition of renal composite end points is required.

Trials in the treatment of SLE need to standardise outcomes and reporting in order that results can contribute to a coherent picture of treatment efficacy and safety.

### Supplementary Information

**Additional file 1.**

**Additional file 2.**

**Additional file 3.**

## Data Availability

The published article contains summarised versions of significant results generated and analysed during this study. A full set of the unedited data presented via forest plots is provided in the supplementary files.

## Abbreviations

AM: Antimalarials
ACR: American College of Rheumatology
APRIL: A proliferation-inducing ligand
AZA: Azathioprine
BAFF: B-cell activating factor
BCMA: B cell maturation antigen
BICLA: BILAG-Based Composite Lupus Assessment
BID: Twice daily
BILAG: British Isles Lupus Assessment Group
BlyS: B-lymphocyte stimulator
CYC: Cyclophosphamide
eGFR: Estimated glomerular filtration rate (measured in ml per minute per 1.73 m^2^)
EULAR: European League Against Rheumatism
HPF: High powered field
IFN: Interferon
IV: Intravenous
JAK: Janus kinase
MMF: Mycophenolate mofetil
MFS: Mycophenolate sodium
MTX: Methotrexate
NSAIDS: Nonsteroidal anti inflammatory drugs
QD: Once daily
RPGN: Rapidly progressing glomerulonephritis
RBC: Red blood cell
SC: Subcutaneous
SLE: Systemic lupus erythromatosus
SLEDAI: Systemic Lupus Erythematosus Disease Activity Index
SLICC: Systemic Lupus International Collaborating Clinics
SRI: Systemic Lupus Erythematosus Responder Index
TACI: Transmembrane activator and calcium modulator and cySM101clophylin ligand interactor
UPCR: Urine protein to creatinine ratio
WBC: White blood cell
