# Supplementary material for: Safety and efficacy of biological agents in the treatment of Systemic Lupus Erythematosus (SLE)
Source: BMC Rheumatol. 2023 Oct 9;7:37. doi: 10.1186/s41927-023-00358-3 (PMC10561476; doi:10.1186/s41927-023-00358-3)
Supplement: Supplementary file 1 — Additional file 1. [file 41927_2023_358_MOESM1_ESM.pdf]

Table of Contents

PRISMA checklist 2
Search methods 8
Excluded studies 8
Premature termination/insufficient data 8
Subanalysis/abstract of another paper 9
Not fulfilling study criteria 10
Groups of drugs without significant results 11
Anti-dsDNA complexing 11
Abetimus 11
Selective Janus kinase (JAK)1 and JAK2 inhibitor 11
Baricitinib 11
Bruton’s tyrosine kinase inhibitor 12
Evobrutinib 12
Fenebrutinib 12
High-affinity cereblon ligand 13
CC-220/Iberdomide 13
Tolerogenic peptides 13
Edratide 13
Anti CD22 monoclonal antibody 14
Epratuzumab 14
Anti-interleukin (IL) 6 antibody 14
PF-04236921 14
Vobarilizumab 15

Anti Interleukin-10 monoclonal antibody 15
BT063 15
P140 peptide 16
Lupuzor 16
Recombinant, soluble human FcγRIIb 16
SM101 16

PRISMA checklist

		Reporting Item	Page Number
**Title**			
Title	#1	Identify the report as a systematic review	Abstract title
**Abstract**			
Abstract	#2	Report an abstract addressing each item in the PRISMA 2020 for Abstracts checklist	Abstract completed
**Introduction**			
Background/rationale	#3	Describe the rationale for the review in the context of existing knowledge	Introduction section
Objectives	#4	Provide an explicit statement of the objective(s) or question(s) the review addresses	Introduction section
**Methods**			
Eligibility criteria	#5	Specify the inclusion and exclusion criteria for the review and how studies were grouped for the syntheses	Methods section

Information sources	#6	Specify all databases, registers, websites, organisations, reference lists, and other sources searched or consulted to identify studies. Specify the date when each source was last searched or consulted	Methods section, PRISMA flow diagram
Search strategy	#7	Present the full search strategies for all databases, registers, and websites, including any filters and limits used	Appendix, search methods
Selection process	#8	Specify the methods used to decide whether a study met the inclusion criteria of the review, including how many reviewers screened each record and each report retrieved, whether they worked independently, and, if applicable, details of automation tools used in the process	Methods section
Data collection process	#9	Specify the methods used to collect data from reports, including how many reviewers collected data from each report, whether they worked independently, any processes for obtaining or confirming data from study investigators, and, if applicable, details of automation tools used in the process	Methods section
Data items	#10 a	List and define all outcomes for which data were sought. Specify whether all results that were compatible with each outcome domain in each study were sought (for example, for all measures, time points, analyses), and, if not, the methods used to decide which results to collect	Supplementary tables
Data items	#10 b	List and define all other variables for which data were sought (such as participant and intervention characteristics, funding sources). Describe any assumptions made about any missing or unclear information	Methods section

Study risk of bias assessment	#11	Specify the methods used to assess risk of bias in the included studies, including details of the tool(s) used, how many reviewers assessed each study and whether they worked independently, and, if applicable, details of automation tools used in the process	Yes, quality of study using Revman risk of bias tool
Effect measures	#12	Specify for each outcome the effect measure(s) (such as risk ratio, mean difference) used in the synthesis or presentation of results	Yes. Statistical assessment
Synthesis methods	#13a	Describe the processes used to decide which studies were eligible for each synthesis (such as tabulating the study intervention characteristics and comparing against the planned groups for each synthesis (item #5))	Studies included/reported statment
Synthesis methods	#13b	Describe any methods required to prepare the data for presentation or synthesis, such as handling of missing summary statistics or data conversions	Outcomes didn't measure continuous data
Synthesis methods	#13c	Describe any methods used to tabulate or visually display results of individual studies and syntheses	Grade tables and forest plots
Synthesis methods	#13d	Describe any methods used to synthesise results and provide a rationale for the choice(s). If meta-analysis was performed, describe the model(s), method(s) to identify the presence and extent of statistical heterogeneity, and software package(s) used	Forest plots
Synthesis methods	#13e	Describe any methods used to explore possible causes of heterogeneity among	Subgroup analysis not done

		study results (such as subgroup analysis, meta-regression)	
Synthesis methods	#13f	Describe any sensitivity analyses conducted to assess robustness of the synthesised results	Sensitivity analyses not done
Reporting bias assessment	#14	Describe any methods used to assess risk of bias due to missing results in a synthesis (arising from reporting biases)	Methods<br>Appendix: Risk of bias graph/summary
Certainty assessment	#15	Describe any methods used to assess certainty (or confidence) in the body of evidence for an outcome	Appendix: Summary of findings; Grade tables
**Results**			
Study selection	#16a	Describe the results of the search and selection process, from the number of records identified in the search to the number of studies included in the review, ideally using a flow diagram (http://www.prisma-statement.org/PRISMAStatement/FlowDiagram)	Appendix, Prisma flow diagram
Study selection	#16b	Cite studies that might appear to meet the inclusion criteria, but which were excluded, and explain why they were excluded	Appendix, Excluded papers
Study characteristics	#17	Cite each included study and present its characteristics	Table 1 Study characteristics
Risk of bias in studies	#18	Present assessments of risk of bias for each included study	Appendix; Risk of bias graph/summary
Results of individual studies	#19	For all outcomes, present for each study (a) summary statistics for each group (where appropriate) and (b) an effect estimate and	Forest plots

		its precision (such as confidence/credible interval), ideally using structured tables or plots	
Results of syntheses	#20a	For each synthesis, briefly summarise the characteristics and risk of bias among contributing studies	Present in each individual drug write up and Appendix, risk of bias table/graph
Results of syntheses	#20b	Present results of all statistical syntheses conducted. If meta-analysis was done, present for each the summary estimate and its precision (such as confidence/credible interval) and measures of statistical heterogeneity. If comparing groups, describe the direction of the effect	Appendix, Forest plots
Results of syntheses	#20c	Present results of all investigations of possible causes of heterogeneity among study results	Described in individual drugs when available, forest plot data
Results of syntheses	#20d	Present results of all sensitivity analyses conducted to assess the robustness of the synthesised results	Sensitivity analysis not performed
Risk of reporting biases in syntheses	#21	Present assessments of risk of bias due to missing results (arising from reporting biases) for each synthesis assessed	Appendix, risk of bias tables
Certainty of evidence	#22	Present assessments of certainty (or confidence) in the body of evidence for each outcome assessed	Appendix, summary of finding GRADE tables
**Discussion**			
Results in context	#23a	Provide a general interpretation of the results in the context of other evidence	Comparison with other systematic reviews section

Limitations of included studies	#23 b	Discuss any limitations of the evidence included in the review	Difficulty with outcome measures section
Limitations of the review methods	#23 c	Discuss any limitations of the review processes used	Difficulty with outcome measures section
Implications	#23 d	Discuss implications of the results for practice, policy, and future research	Recommendations for future research section
**Other information**			
Registration and protocol	#24 a	Provide registration information for the review, including register name and registration number, or state that the review was not registered	Not registered
Registration and protocol	#24 b	Indicate where the review protocol can be accessed, or state that a protocol was not prepared	Protocol not prepared
Registration and protocol	#24 c	Describe and explain any amendments to information provided at registration or in the protocol	Refer 24b
Support	#25	Describe sources of financial or non-financial support for the review, and the role of the funders or sponsors in the review	No supports involved
Competing interests	#26	Declare any competing interests of review authors	No conflict of interests
Availability of data, code, and other materials	#27	Report which of the following are publicly available and where they can be found: template data collection forms; data extracted from included studies; data used	References

for all analyses; analytic code; any other
materials used in the review

Search methods

This review was conducted according to the PRISMA check list. MEDLINE (through Pubmed), EMBASE, the Cochrane library, and Clinicaltrials.gov, Australianclinicaltrials.gov, ANZCTR.gov and WHO International Clinical Trials Regsitry Platform were searched on 20 May 2021, using the search terms (Medical Subject Heading (MeSH), EMTREE and/or free text) for (I) Clinical trials (II) Randomized control trials and (III) systemic lupus erythematosus (systemic lupus, lupus, and SLE) (IV) Biologic/Biological agents (monoclonal antibodies,). Search terms within (I) and (II) were combined using 'OR', and both groups of search terms combined with 'AND' (III and IV). Search results were limited to human clinical trials in English, published within 15 years, for which full-text articles were available for appraisal. All MeSH and EMTREE terms were exploded. A grey literature search was conducted, all data collected was initially completed by 30 May 2021.

Excluded studies

Premature termination/insufficient data

Ginzler Atacicept 2012 - Prematurely terminated witin a week due to marked drop in IgG, no other useful data extractable from paper

Kahl 2016 - premature termination at interim analysis due to futility

NCT00447265 Etanercept 2013 - premature termination after 1 patient in Etanercept group, safety investigators decided risk-benefit ratio not acceptable based on data looking at other Etanercept trials

NCT01085097 Laquinimod sodium - no published data

NCT01499355 BIIB023 - study terminated, no data available

NCT01845740 Milatuzumab - phase 1b study, no data available

NCT02711813 Toralizumab - study terminated, no data available

NCT02955615 ILT-101 - no published data ongoing study

NCT03451422 AMG592 - Phase 1b, ongoing study

Subanalysis/abstract of another paper

Merrill 2012 (Belimumab) - Long term follow up study of Wallace 2009 Belimumab study. extension study, patients no longer randomised as they entered an open label study

Ginzler 2014 (Belimumab)- Long term follow up of Wallace 2009 study at 4 years, only follows intervention arm but not placebo/control arm

Tanaka 2017 (Belimumab) - Subset of Zhang 2018 paper

Aranow 2018 - Abstract of Atisha Fregoso 2021 CALIBRATE study

Doria 2018 (Belimumab) - Subset of Stohl 2017 Belimumab paper

Furie 2018 (Belimumab) - Long term study of BLISS-76 paper , only follows up intervention arm with Belimumab without comparison placebo/control arm

Furie 2020 NOBILITY (Obinutuzumab)- 2nd year extension data from Furie 2019 paper, data amalgamated into Furie 2019 outcomes

Rovin 2020 (Obinutuzumab) - Subset of Furie 2019 , looking at response at 76 weeks depending on B cell depletion status

Vital 2020 (Obinutuzumab)- Subset of Furie 2019 Nobility study. Looks to be a duplicate of Rovin 2020 abstract as well

Not fulfilling study criteria

Arienger 2009 - Follow up of patients on infliximab only, not RCT

Baker 2020 - Not a randomised trial, compared either filgotinib or lanraplenib, no placebo arm

Decreux 2016- Testing only on patient serum samples, not a clinical trial. Not randomised/no control arm/non clinical trial

Dooley 2016 - Voclosporin not a biologic

Fernandez 2006 - Rapamycin not a biologic agent

Furie 2001 – Beyond data collection period of 15 years

Hasni 2019 - Phase 1b study, abstract only

Llorente 2000 - Study on sle patients, but no controls, not a clinical trial

Masoud 2018 - Case report series of Ofatumumab, not a randomised trial

Merrill 2018 XmAb®5871 - No data published

NCT02847598 BIIB059 - studying cutaneous lupus with/without other lupus manifestations. no data yet as well

NCT03159936 Tofacitinib - study only discoid lupus patients only

Reddy Obitunuzumab 2017 - not clinical trial, study performed only on patient blood samples to study B cell cytotoxicity

Rovin 2018 – voclosporin not a biologic

Shi R Lulizumab 2019 - Pharmacokinetic,pharmacodynamic, not phase 2/3 RCT study, and in healthy subjects without SLE

Streicher 2018 - not a clinical trial, studying skin biopsy samples from other trials, also includes disease other than SLE in study (COPD)

Groups of drugs without significant results

Anti-dsDNA complexing

Anti-dsDNA antibodies are one of the measured autoantibodies in SLE and form part of its diagnostic criteria. The production of anti-dsDNA antibodies in SLE are likely related to impaired tolerance to immunogenic self-DNA in SLE patients, which may mediate disease activity. Abetimus is currently the only drug in this class.

Abetimus

Abetimus is composed of 4 identical strands of dsDNA with specificity for anti-dsDNA antibodies. It is thought to function by forming drug-antibody complexes, reducing circulating anti-dsDNA antibody levels and tolerizing anti-dsDNA specific B cells.

One study consisting of 317 patients addressed the use of Abetimus in SLE: Cardiel 2008 (33). A single dose of Abetimus 100mg IV weekly for up to 22 months was studied.

The main outcome studied was time to a renal flare, which was not an outcome analysed in this review, though there was no significant difference between the Abetimus and placebo group Abetimus usage did not increase any of the adverse events studied.

Selective Janus kinase (JAK)1 and JAK2 inhibitor

JAK1 and JAK2 are tyrosine kinases that mediate intracellular pathway signalling of Type 1 and Type II cytokine receptors including interleukins and IFNs, both of which are raised in SLE, including IL-6, IL-12, IL-23, and Type 1 IFN which are being studied as potential therapeutic targets. Targeting the JAK/STAT pathway may allow suppression of these cytokines.

Baricitinib

Baricitinib is an orally administered selective and reversible inhibitor of JAK1 and JAK2.

One study addressed Baricitib use in SLE(34) recruiting 3141 patients. Patients with lupus nephritis and CNS lupus were excluded. Doses of Barictinib PO 2mg and 4mg daily were studied. Comparator

treatments included continuation of previous maintenance regimens using antimalarials, AZA, MMF, MTX and steroids.

The main study outcome was SRI 4 at 24 weeks. Pooled Baricitinib groups of 2mg and 4mg did not increase SRI 4 at 24 weeks (RR 1.22, CI 0.96 to 1.53, P=0.10), though the 4mg group alone achieved significance, (RR 1.35, CI 1.06 to 1.73, P=0.02).

There were no significant differences in safety outcomes.

Bruton's tyrosine kinase inhibitor

BTK is expressed in B and myeloid cells, and functions as a intracellular signalling molecule involved B cell development, survival and antigen presentation and antibody production by B cell antigen receptors. BTK positive cells are increased in peripheral blood SLE patients, with higher anti-dsDNA antibody levels, proteinuria, SLEDAI scores and lower C3 levels(35).

Evobrutinib

Evobrutinib is an inhibitor of Bruton's tyrosine kinase which prevents B cell activation.

One study addressed the use of Evobrutinib in SLE: Wallace 2019 (36)which consisted of 469 patients. Patients with CNS lupus were excluded. Evobrutinib dosages studied included 25mg QD, 75mg QD and 60mg BID PO. Comparator treatments included continuation of previous maintenance regimens.

Main study outcomes were SRI 4, SRI 6 and BICLA at 52 weeks, none which achieved significance (p=0.26, 0.36 and 0.97)

Infectious adverse events were not fully reported, but other safety outcomes including death did not achieve significance.

Fenebrutinib

Fenebrutinib is an orally administrated, reversible inhibitor of Bruton's tyrosine kinase.

One study addressed the use of Fenebrutinib in SLE: Isenberg 2019 (36), which consisted of 260 patients. Patients with CNS and renal involvement were excluded. Evobrutinib dosages studied included 150mg QD, and 200mg BID. Comparator treatments included continuation of previous maintenance regimens using antimalarials, AZA, MMF, MTX and steroids.

Main study outcomes studied were SRI 4, SRI 6 and BICLA at 24 weeks and 52 weeks, none which achieved significance (outcomes at 24 weeks: P=0.35, 0.69 and 0.74, at 52 weeks: P=0.30, 0.14 and 0.36)

There were no significant differences in safety outcomes.

High-affinity cereblon ligand

Encoding transcription factors IKZF1 (Ikaros) and to a lesser extent, IKZF3 (Aiolos) polymorphisms are associated with an increased risk of SLE, and higher levels are found in SLE patients. Cullin ring ligase 4-cereblon E3 ubiquitin ligase (CRL4) is an ubiquitin ligase that targets Ikaros and Aiolos for proteosomal degradation. Reductions in Ikaros and Aiolos levels is associated with decrease in B cell plasmablast differentiation, BAFF levels, CD40L induced proliferation of B-cells and IgG secretion. Currently, CC-220/Iberdomide is currently the only drug in this class.

CC-220/Iberdomide

Two studies addressed the use of CC220/Iberdomide in SLE: Gaudy 2017(37) and Merrill 2020(38)

The 2 studies consisted of 330 patients, with Merrill 2020 having 288 patients. Patients with lupus nephritis and CNS lupus were excluded. Dosages of CC-220/Iberdomide are described in study protocols. Comparator treatments included continuation of previous maintenance regimens using steroid and other immunosuppressants.
Main outcomes in Gaudy 2017 were safety outcomes including adverse events and death. Merrill 2020 studied SRI 4 at 24 weeks, which did not improve with use of Iberdomide (RR 1.35, CI 0.98 to 1.88, P=0.07)

CC-220/Iberdomide did not reduce the use of prednisone in Merrill 2020, with proportion of patients with prednisone reduction to ≤10mg/day not achieving significance (RR 0.20, CI 0.02 to 2.09, P=0.18)

Adverse events were increased with CC-220/Iberdomide in Merrill 2020 , (RR 1.45, CI 1.02 to 2.06 P=0.04), with increased urinary tract, upper respiratory infections, influenza and neutropaenia, but not in the pooled data of both studies or Gaudy 2017 (Figure 19). Treatment related adverse events were increased with CC-220/Iberdomide RR1.39, CI 1.02 to 1.90, P=0.04) (Figure 23). There were no significant differences in the other safety outcomes in both studies.

Tolerogenic peptides

Edratide

Edratide is a tolerogenic peptide based on the complementarity-determining region 1 (CDR1) of a human anti-DNA mAb. Murine models treated with Edrateide showed a decrease in IFN-Y, IL-10 and IL-1B, BAFF/BLyS and an increase in CD4 and CD8 regulatory T cells.

One study addressed the used of Edratide in SLE: Urowitz 2015 (39), including 340 patients. Patients with CNS lupus, lupus nephritis and using any immunosuppressants aside from prednisone were excluded. Edratide dosage are described in study protocols. Comparator treatments only allowed for prednisone, antimalarials and NSAIDs.

The main outcomes studied were BILAG and SLEDAI improvement, which were not analysed in this review, though both outcomes were not increased with the use of Edratide compared to placebo.

Edratide use did not increase adverse events and death.

Anti CD22 monoclonal antibody

B cells exclusively express CD22 (Siglec-2), which have been shown to inhibit B-cell receptor signalling. 2 immunoregulatory tyrosine-activating motifs and/or immunoregulatory tyrosine-inhibiting motifs (ITIMs) on CD22 recruit PTP and SHP-1 which when phosphrylated forms a SHIP complex and activation of MAP kinase which regulates cell survival and proliferation. Epratuzumab is the only drug in this class tested in SLE patients.

Epratuzumab

Epratuzumab is a humanized IgG monoclonal antibody against CD22, derived from the murine IgG2 monoclonal antibody.

Three studies addressed the use of Epratuzumab in SLE: Wallace 2013 (ALLEVIATE ½) (42) , Wallace 2013 (EMBLEM) (41) and Clowse 2017 (EMBODY 1/2) (40)

The 3 studies consisted of 1286 patient. EMBLEM was a 12 week study compared to 52 weeks in EMBODY. ALLEVIATE 1/2 was discontinued prematurely due to an interruption in drug supply. Epratuzumab dosages are summarised in the study protocols. Comparator treatments included continuation of previous maintenance regimens using steroid and other immunosuppressants.

Main study outcomes were SRI 4 and BICLA response.

In the pooled data of all the dosages, Epratuzumab did not increase BICLA response, at 12 weeks in EMBLEM (RR 1.90, CI 0.83 to 4.39, P=0.13) and 52 weeks in EMBODY 1 /2 (RR 1.08, CI 0.94 to 1.25, P=0.29)

Epratuzumab use did not increase SRI 4 at 52 weeks (RR 1.05, CI 0.91 to 1.22, P=0.51) in Clowse 2017. There were no significant differences in the reported safety outcomes.

Anti-interleukin (IL) 6 antibody

IL-6 levels are shown to be higher in patients with SLE compared to healthy patients, in active compared to inactive SLE. B cells and T cells in SLE also express higher levels of IL-6. CNS lupus patients are also found to have higher CSF levels of IL-6, and urinary IL-6 elevation correlate with lupus nephritis disease activity and anti-dsDNA titres.

PF-04236921

PF-04236921 is a fully human immunoglobulin G2 monoclonal antibody that binds to IL-6.

One study addressed the use anti-IL-6 antibody in SLE: Wallace 2017 (43) , which consisted of 183 patients. Dosages of 10, 50 and 200mg were studied. Comparator treatments included continuation of

previous maintenance regimens using steroids and other immunosuppressants. The 200mg group was prematurely terminated due to 3 deaths, 2 with pulmonary embolism and another from pulmonary embolism and disseminated tuberculosis. Data from the 200mg group was included in the safety analysis, but not in any efficacy outcomes.

Main study outcomes were SRI 4 and BICLA response at 24 weeks, none which achieved significance (RR 0.97, CI 0.61 to 1.55, P=0.90 and RR 1.37, CI 0.77 to 2.44, P= 0.29) Anti IL-6 antibody usage did not increase the proportion of patients reducing prednisone dosages to ≤7.5mg/day, with reduction ≥25% from baseline (RR 2.65, CI 0.63 to 11.23, P=0.18)

There were no significant differences in the other reported safety outcomes.

Vobarilizumab

Vobarilizumab is an anti IL-6 nanobody that binds to and neutralises human IL-6.

One study addressed the use of Vobarilizumab in SLE: NCT0243789 (44) , consisting of 312 patients. Patients with lupus nephritis and CNS lupus were excluded. Vobarilizumab dosages are summarised in the study protocols. Comparator treatments included continuation of previous maintenance regimens using antimalarials, AZA, MMF, MTX and steroids.

The main study outcome was SRI responses at weeks 24 and 52. Vobarilizumab did not increase SRI 4 to 8 at weeks 24 and 52, with P-values ranging from 0.2 to 1.0. There were no significant differences in the safety outcomes.

Anti Interleukin-10 monoclonal antibody

IL-10 levels are increased in patients with SLE and correlates with disease activity, with the majority being produced monocytes, B lymphocytes and a smaller extent, from T lymphocytes. The constant presence of autoantibodies such as anti-dsDNA may allow for B cells to be continuously primed for IL-10 costimulation, promoting B cell differentiation and further autoantibody production. BT063 is currently the only drug in this class.

BT063

BT063 is a humanised anti IL-10 monoclonal antibody. It has currently only been studied in SLE.

One study addressed the use of BT063 in SLE: NCT02554019 (45) consisting of 36 patients. Patients with active and severe lupus nephritis or neuropsychiatric lupus were excluded. Dosages of 50 and 100mg were studied. Comparator treatments allowed for continuation of previous maintenance regimens.

The main outcomes consisted of safety outcomes. Adverse and serious adverse events, withdrawal due to adverse events and death were not increased with BT063

P140 peptide

P140 peptide containing a phosphoserine residue at position 140 formed from spliceosomal U1-70K small nuclear ribonucloproteins which interacts with the HSC70/Hsp73 protein. In MRL/lpr murine models, P140 decreased the expression and folding of HSC70 chaperone proteins and down regulated lysosomal degradation during autophagic flux, possibly decreasing the presentation of self antigens to autoreactive T cells.

Lupuzor

2 studies addressed the use of Lupuzor in SLE: Zimmer 2013 (46), and Wallace 2019 (47) (NCT02504645, unpublished data) consisting of 351 patients. Lupuzor dosages are listed in the study protocols. Comparator treatments included continuation of previous maintenance regimens using antimalarials, AZA, MMF, MTX and steroids.

The main outcomes studied was SRI 4.

Lupuzor did not increase SRI 4 at week 24, (RR 1.11, CI 0.82 to 1.52, P=0.50) in Zimmer 2013 and SRI 4 at 52 weeks in Wallace 2019 (RR 1.18, CI 0.88 to 1.57, P=0.26)

Lupuzor usage did not increase death, serious adverse events and withdrawal due to adverse events.

Recombinant, soluble human FcyRIIb

Immune complexes produced in autoimmune diseases are able to activate FcyRs which stimulate secretion of inflammatory cytokines such as TNF-alpha and IL-6. FcyRIIb is an inhibitory Fc receptor and has been shown to decrease inflammatory cytokines and IgG autoantibodies in murine models for inflammatory arthritis.

SM101

SM101 is a recombinant soluble FcyIIb receptor which binds to the Fc part of immune complexes, inhibiting the binding of immune complexes to cell-standing Fcg receptors. It was previously studied in immune thrombocytopenic purpura but not currently indicated in its treatment.

One study addressed the use SM101 in SLE: Tillmans 2014 (48) which consisted of 51 patient. Patients with lupus nephritis were included, though the class/severity were not stated. SM101 dosages of 6 and 12mg/kg were studied. Comparator treatments included continuation of AZA, MMF and steroids.

The main outcome studied was SRI 4 at 24 weeks, which was not increased with SM101 (RR 2.06, CI 0.55 to 7.69, P= 0.28). No safety data was reported in this small study.
