# Supplementary material for: Safety and efficacy of biological agents in the treatment of Systemic Lupus Erythematosus (SLE)
Source: BMC Rheumatol. 2023 Oct 9;7:37. doi: 10.1186/s41927-023-00358-3 (PMC10561476; doi:10.1186/s41927-023-00358-3)
Supplement: Supplementary file 3 — Additional file 3. [file 41927_2023_358_MOESM3_ESM.docx]

[Figure 1 SRI 4 at 24 weeks 4](#_Toc145354993)

[Figure 2 SRI 4 at 52 weeks 5](#_Toc145354994)

[Figure 3 SRI 5 at 24 weeks 6](#_Toc145354995)

[Figure 4 SRI 5 at 52 weeks 6](#_Toc145354996)

[Figure 5 SRI 6 at 24 weeks 7](#_Toc145354997)

[Figure 6 SRI 6 at 52 weeks 8](#_Toc145354998)

[Figure 7 SRI 7 at 24 weeks 8](#_Toc145354999)

[Figure 8 SRI 7 at 52 weeks 9](#_Toc145355000)

[Figure 9 SRI 8 at 24 weeks 9](#_Toc145355001)

[Figure 10 SRI 8 at 52 weeks 10](#_Toc145355002)

[Figure 11 BICLA at 24 weeks 10](#_Toc145355003)

[Figure 12 BICLA at 52 weeks 11](#_Toc145355004)

[Figure 13 Complete and partial renal remission at 1 year 12](#_Toc145355005)

[Figure 14 Complete and partial renal remission at 2 years 12](#_Toc145355006)

[Figure 15 Change in prednisone dosages to ≤10mg/day 13](#_Toc145355007)

[Figure 16 Change in prednisone dosages to ≤7.5mg and >25% reduction from baseline dosage 14](#_Toc145355008)

[Figure 17 Adverse events 16](#_Toc145355009)

[Figure 18 Serious adverse events 18](#_Toc145355010)

[Figure 19 Death 20](#_Toc145355011)

[Figure 20 Infectious adverse events 22](#_Toc145355012)

[Figure 21 Serious infectious adverse events 24](#_Toc145355013)

[Figure 22 Grade 3 or higher infectious events 24](#_Toc145355014)

[Figure 23 Infusion related adverse events 25](#_Toc145355015)

[Figure 24 Withdrawal due to adverse events 27](#_Toc145355016)

[Figure 25 Withdrawal due to serious adverse events 27](#_Toc145355017)

[Figure 26 Treatment related adverse events 28](#_Toc145355018)

[Figure 27 Serious treatment related adverse events 29](#_Toc145355019)


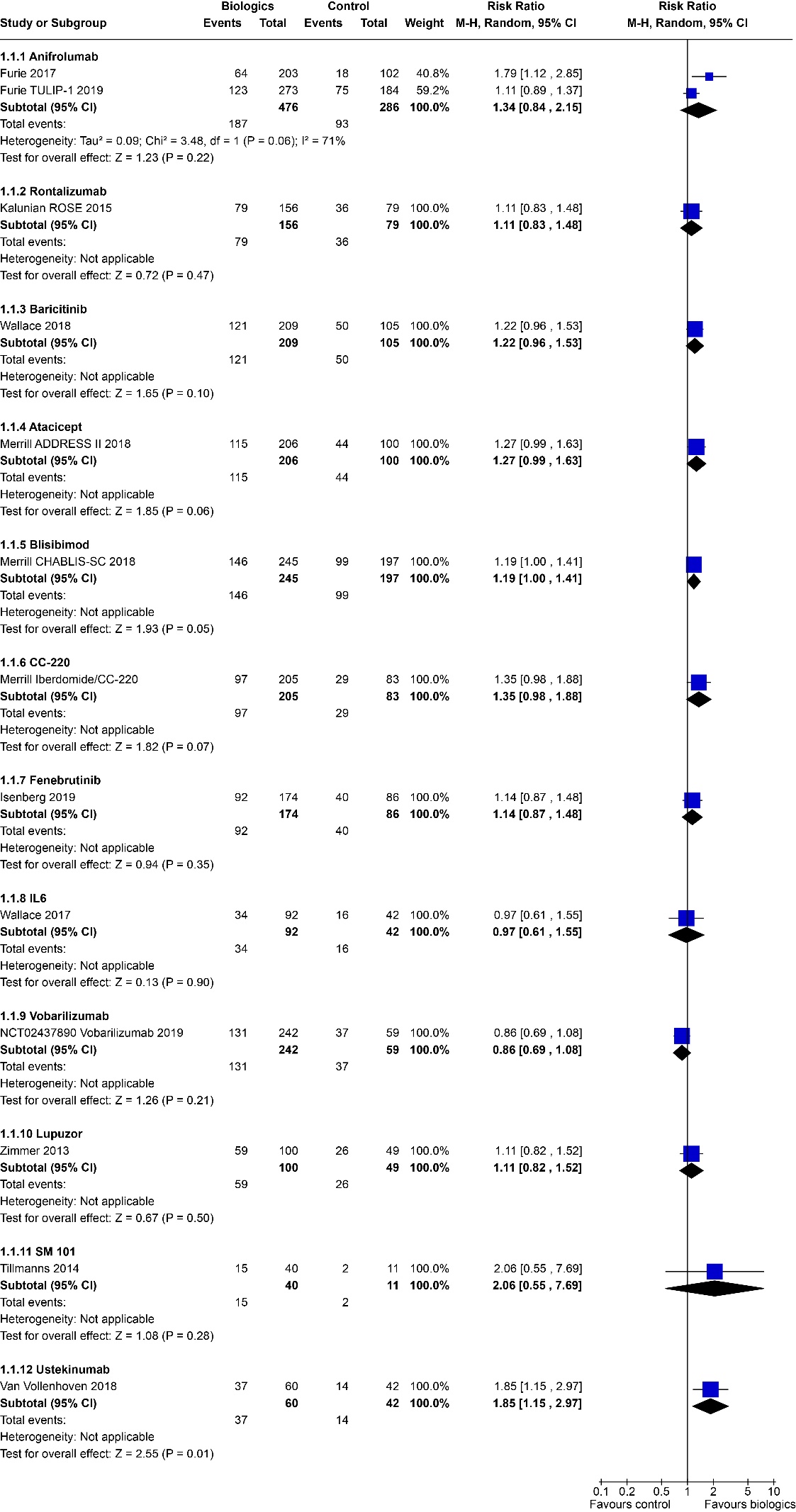


Figure 1 SRI 4 at 24 weeks


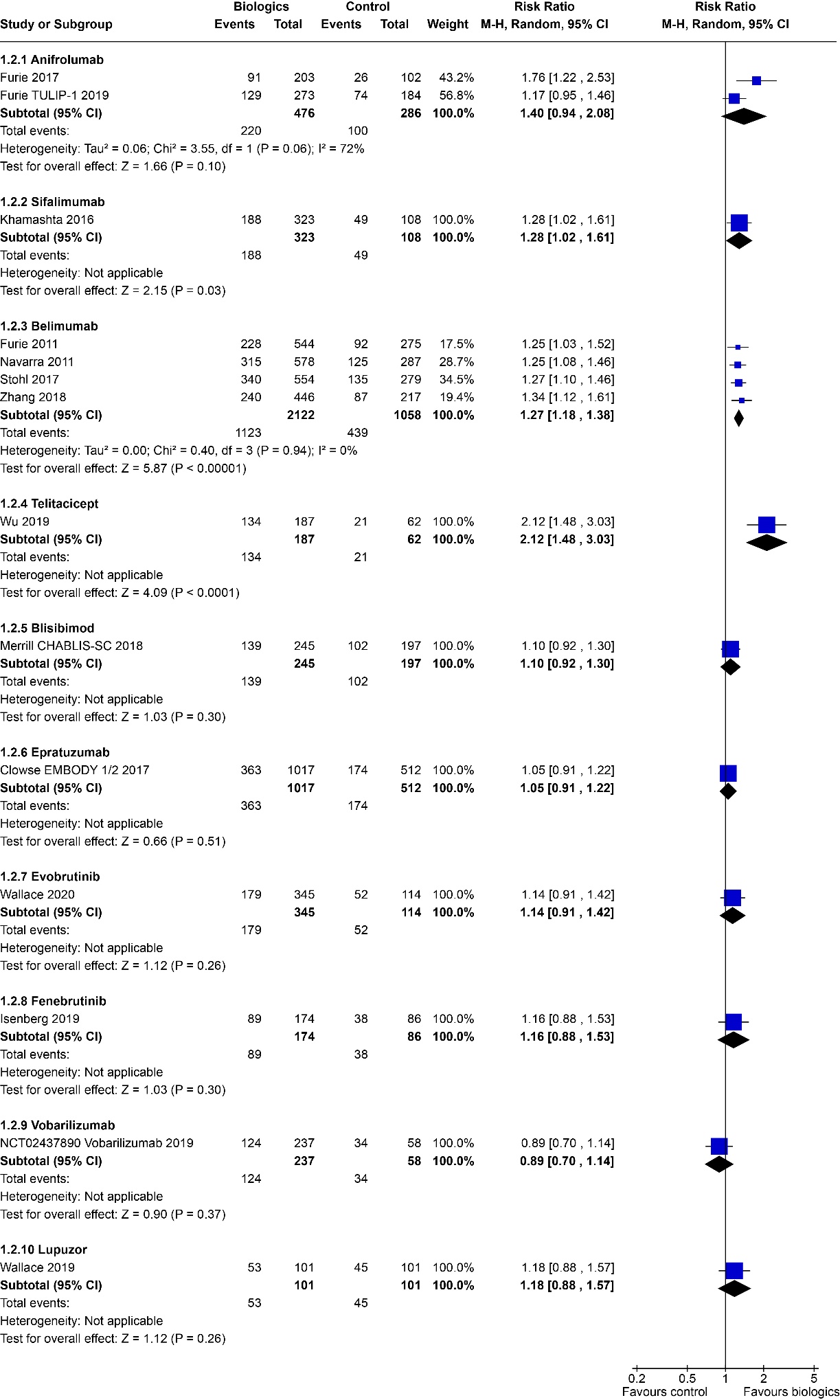


Figure 2 SRI 4 at 52 weeks


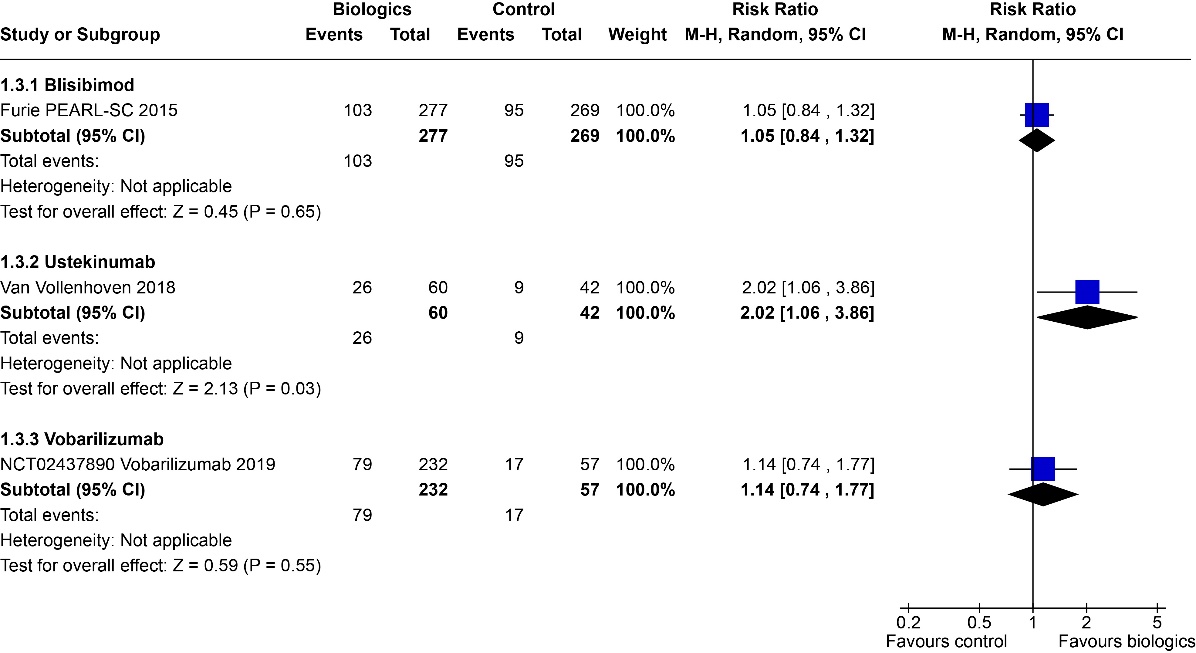


Figure 3 SRI 5 at 24 weeks


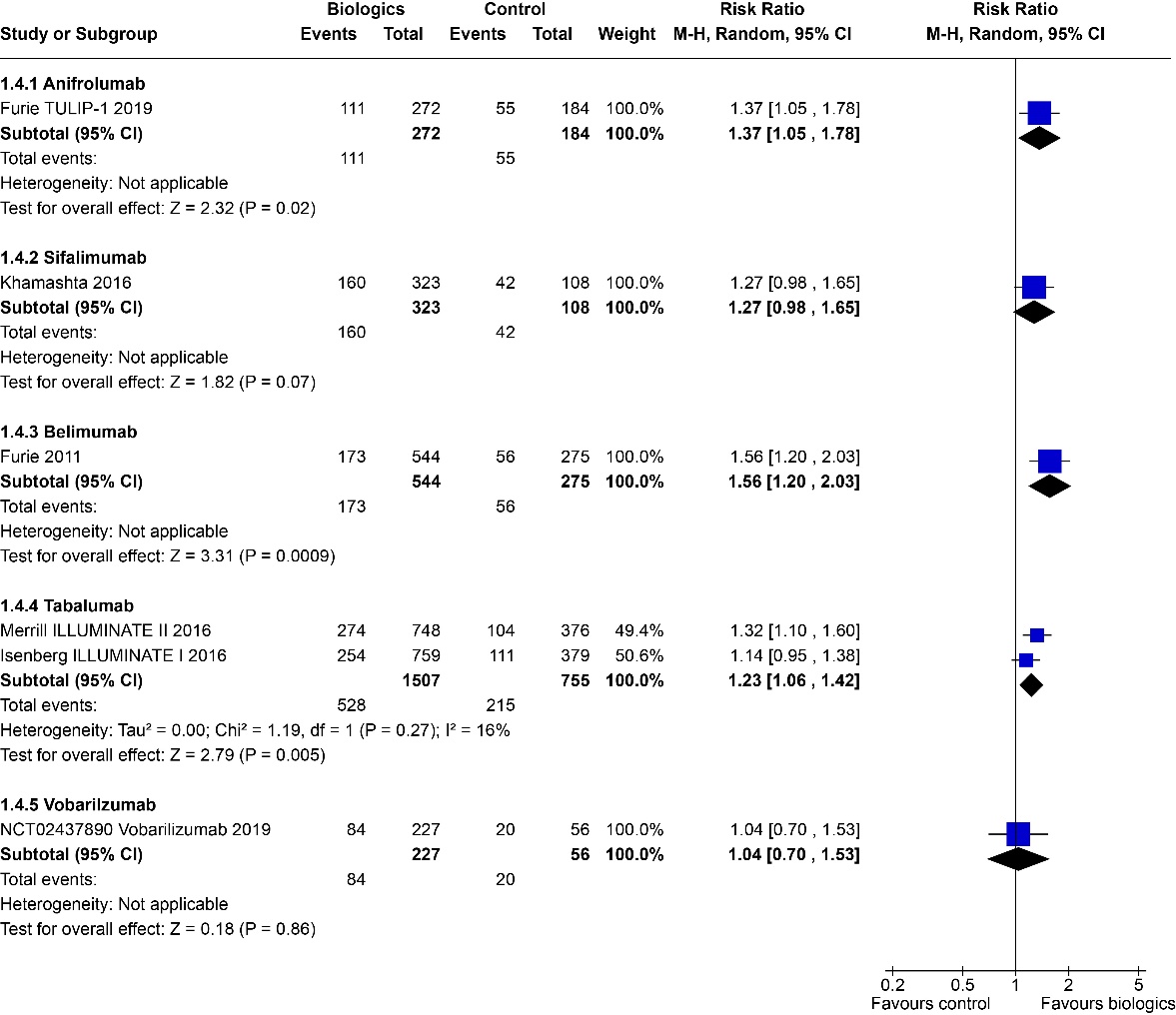


Figure 4 SRI 5 at 52 weeks


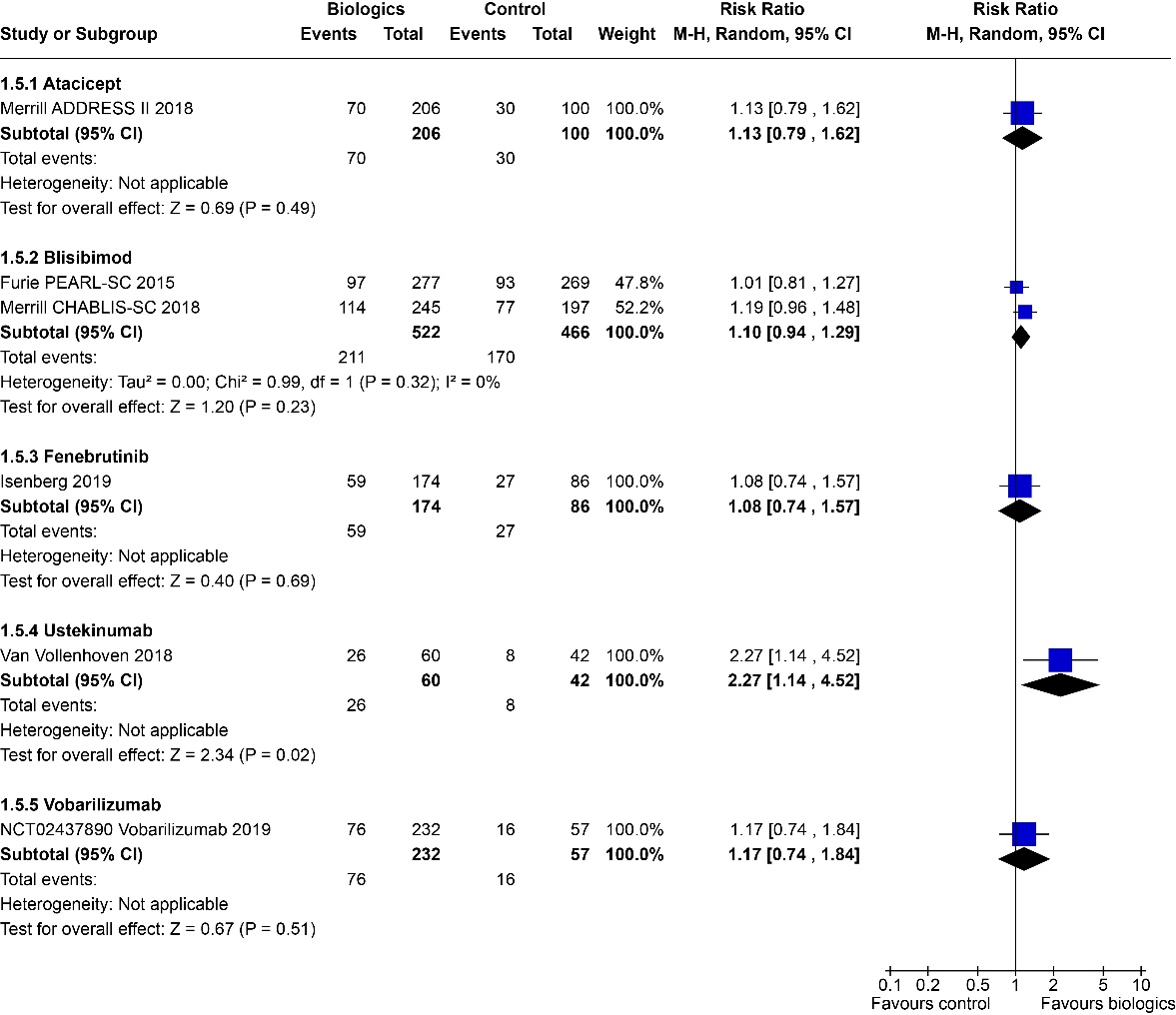


Figure 5 SRI 6 at 24 weeks


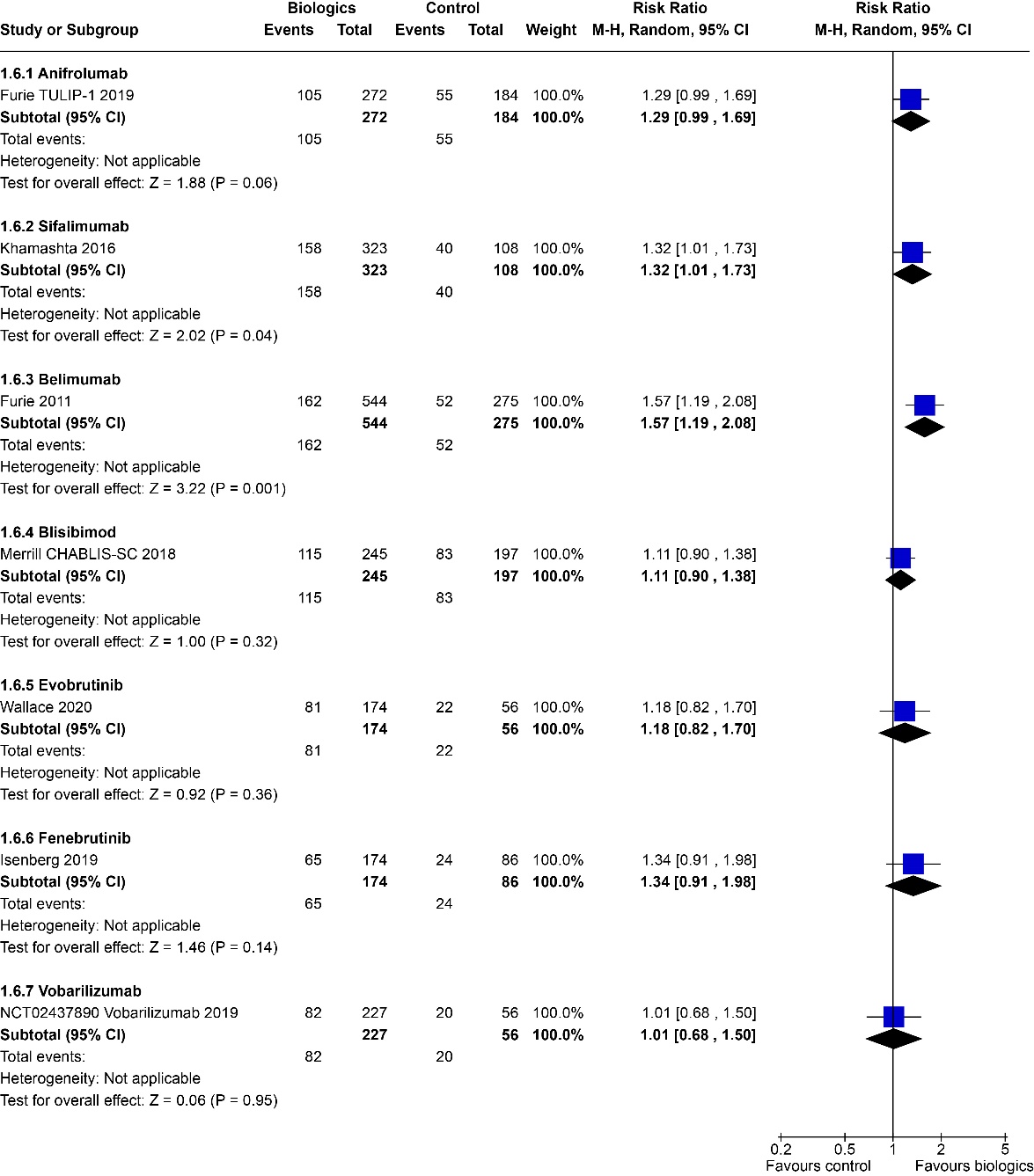


Figure 6 SRI 6 at 52 weeks


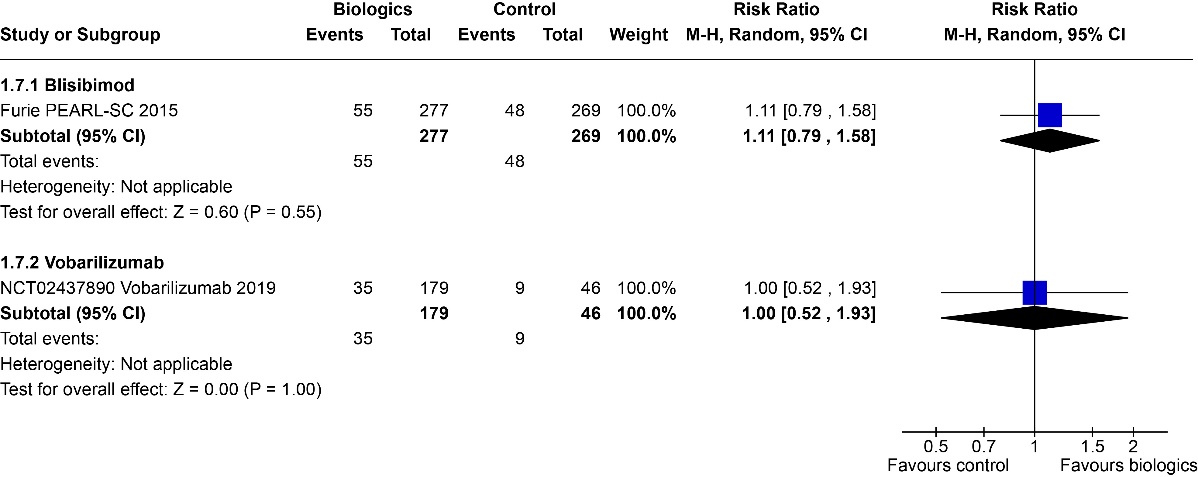


Figure 7 SRI 7 at 24 weeks


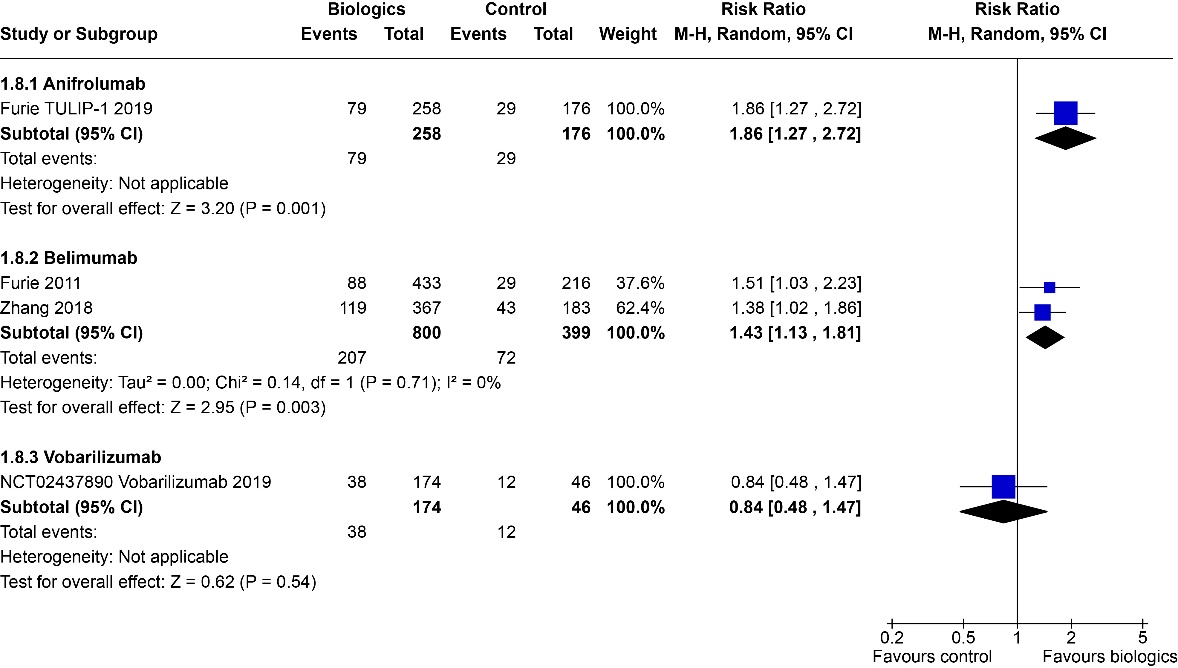


Figure 8 SRI 7 at 52 weeks


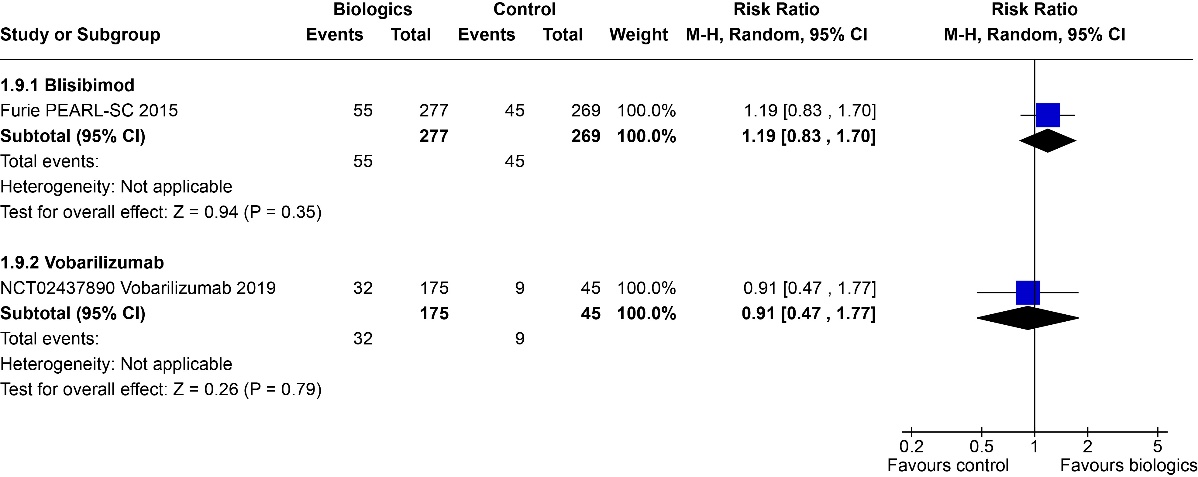


Figure 9 SRI 8 at 24 weeks


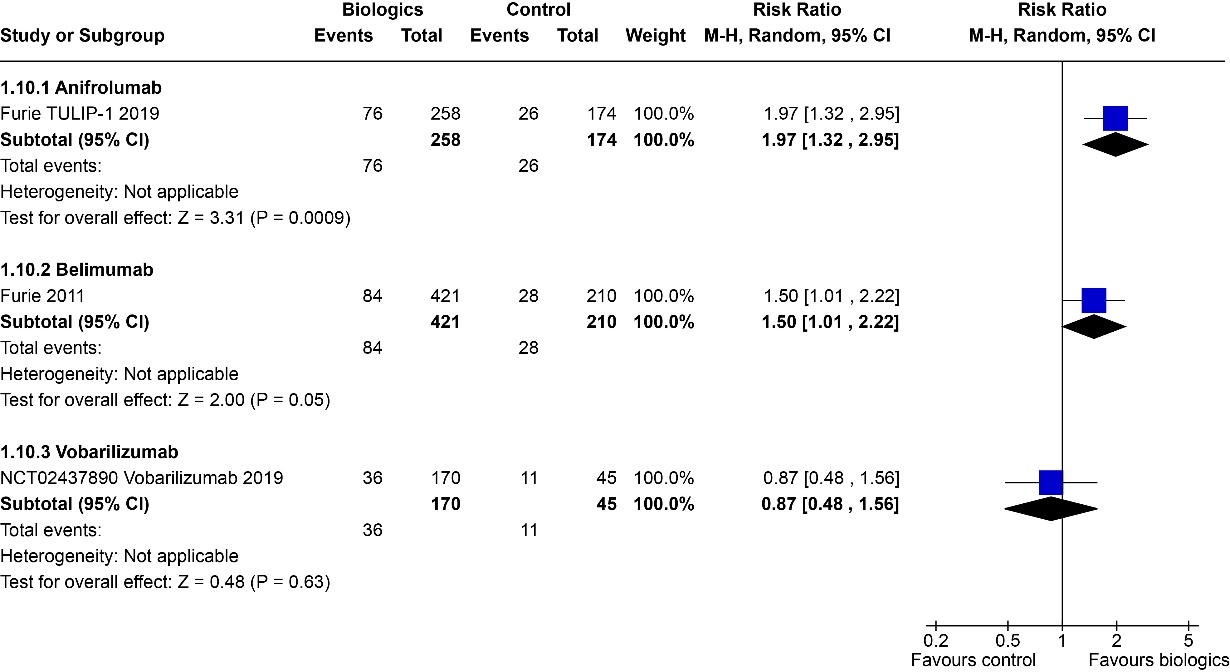


Figure 10 SRI 8 at 52 weeks


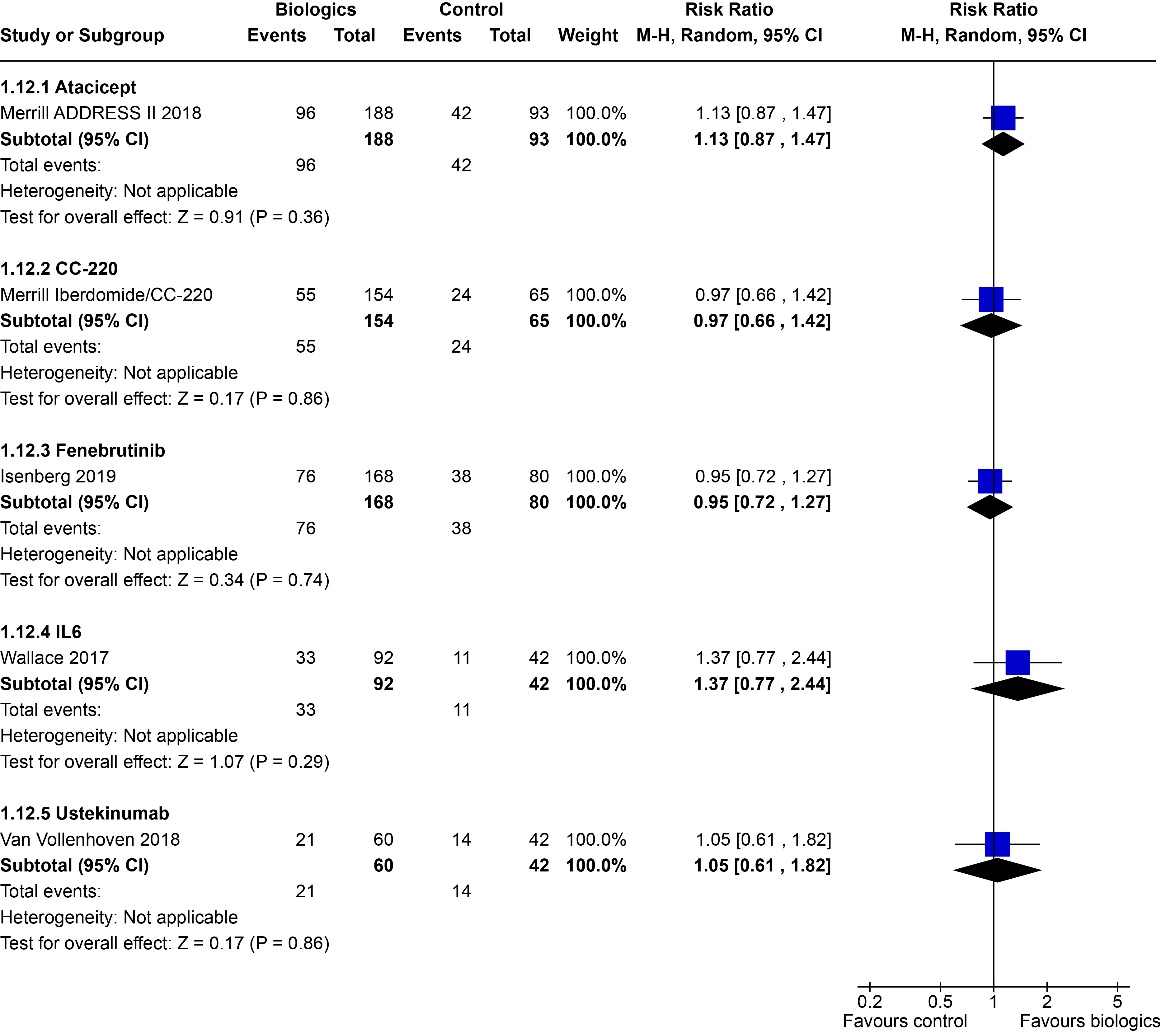


Figure 11 BICLA at 24 weeks


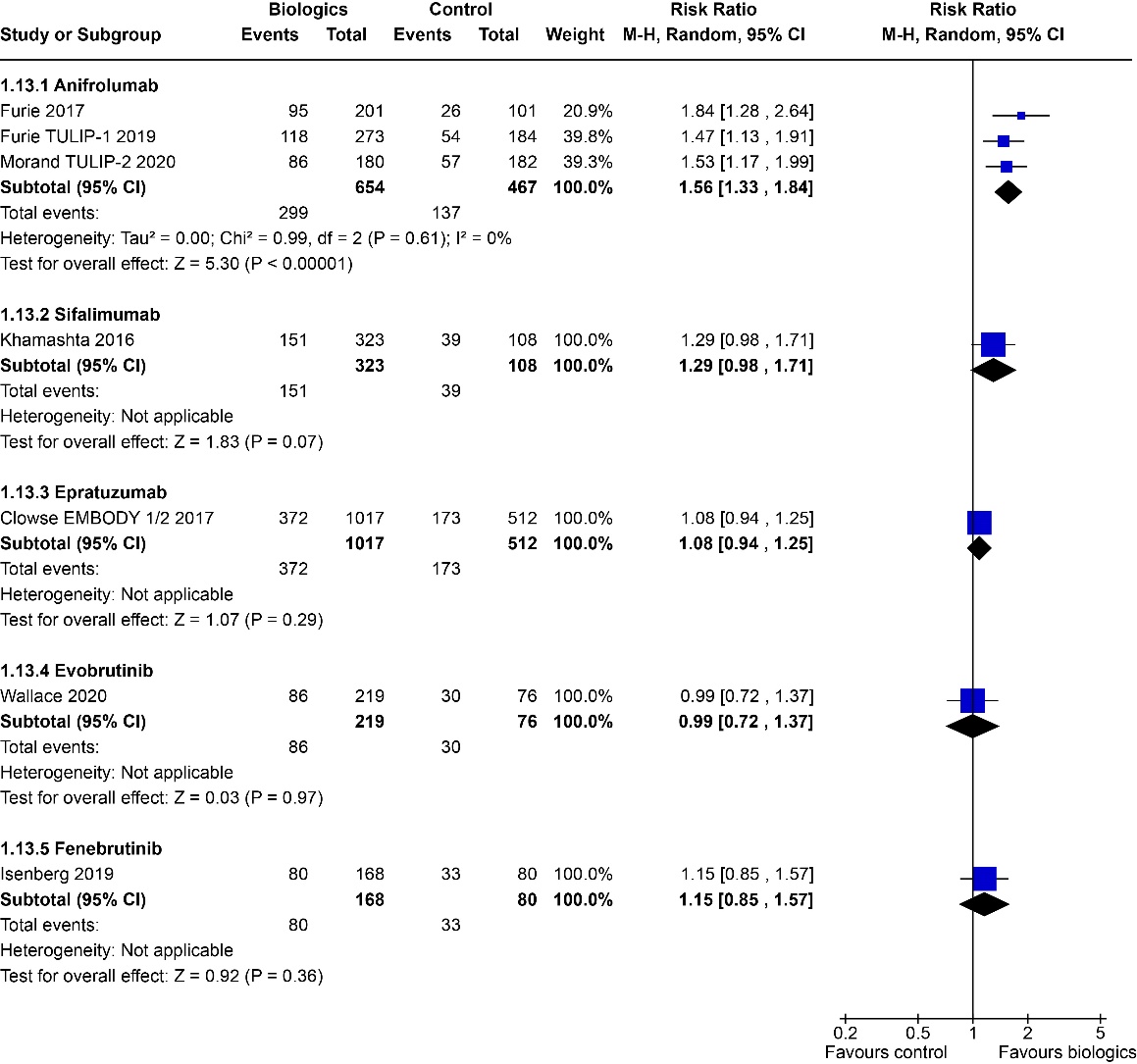


Figure 12 BICLA at 52 weeks


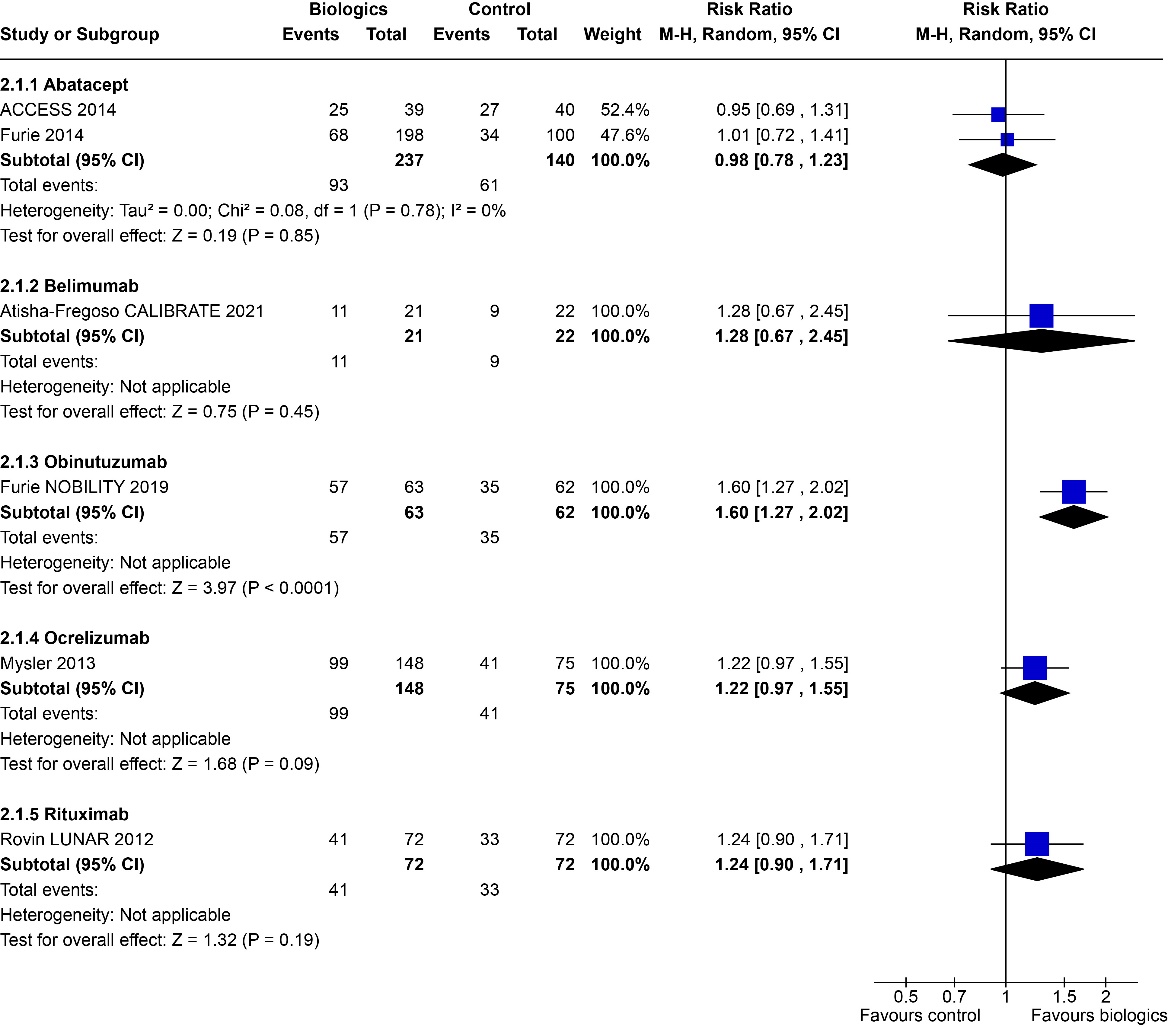


Figure 13 Complete and partial renal remission at 1 year


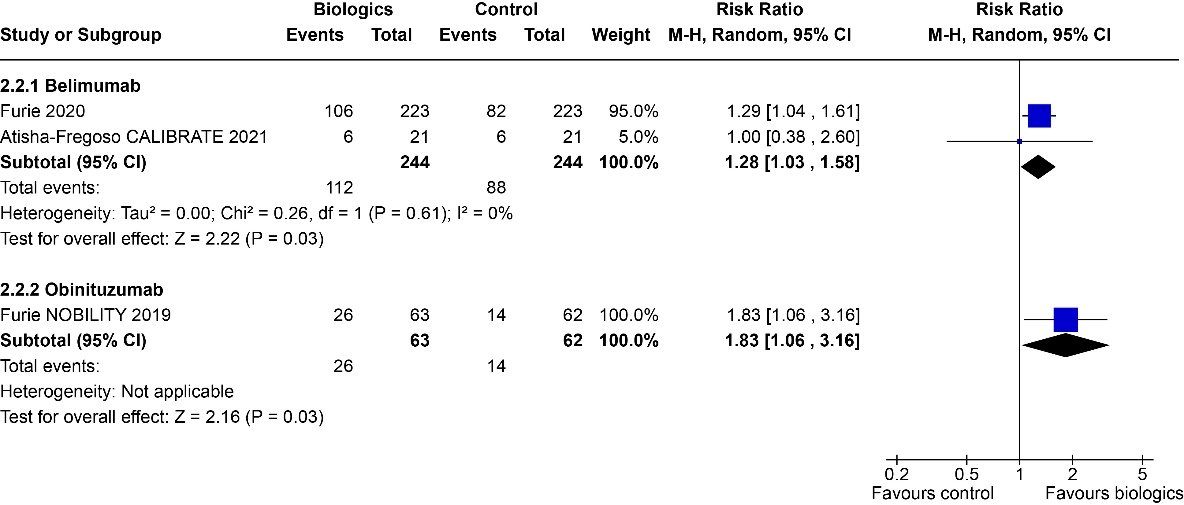


Figure 14 Complete and partial renal remission at 2 years


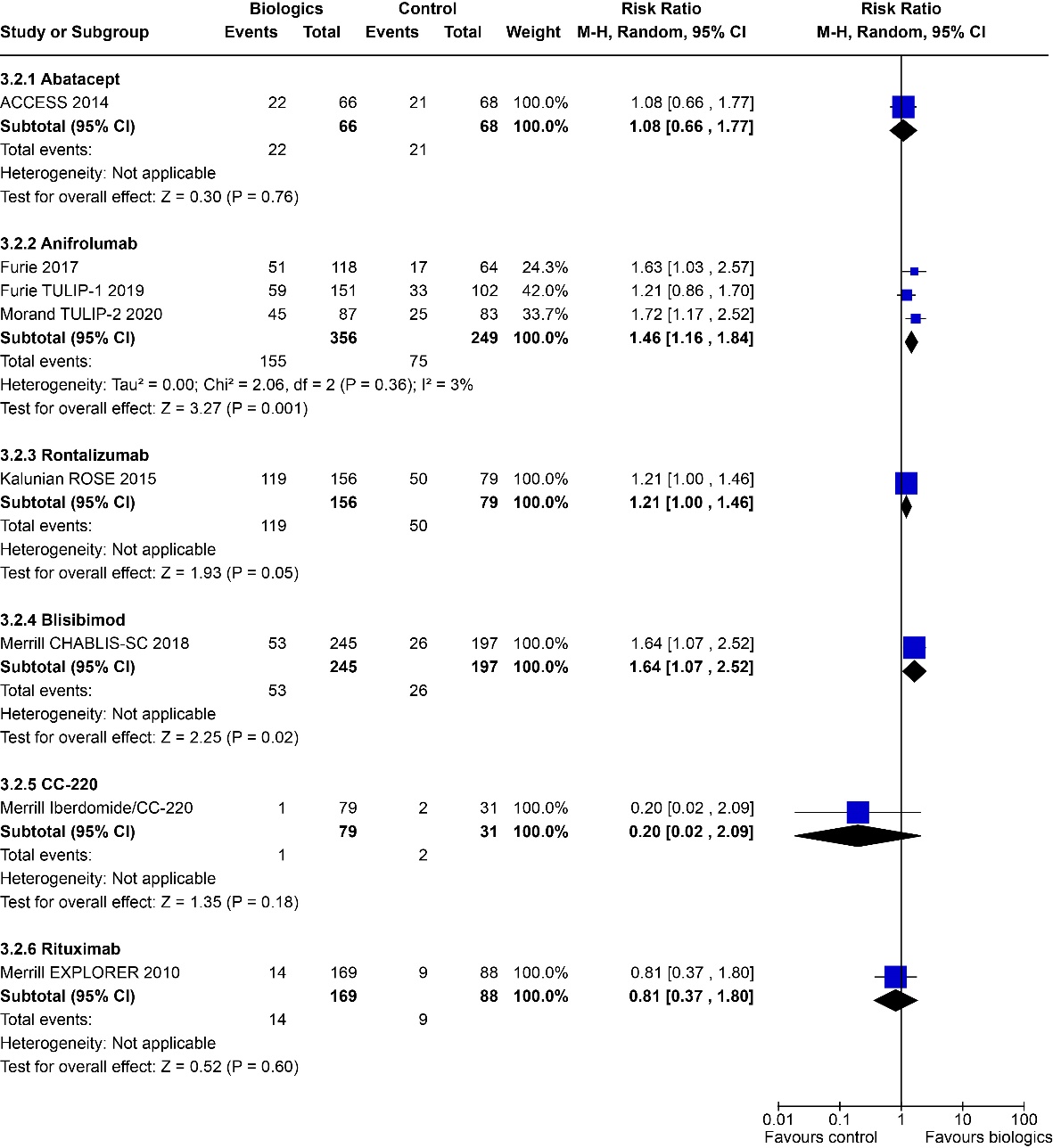


Figure 15 Change in prednisone dosages to ≤10mg/day


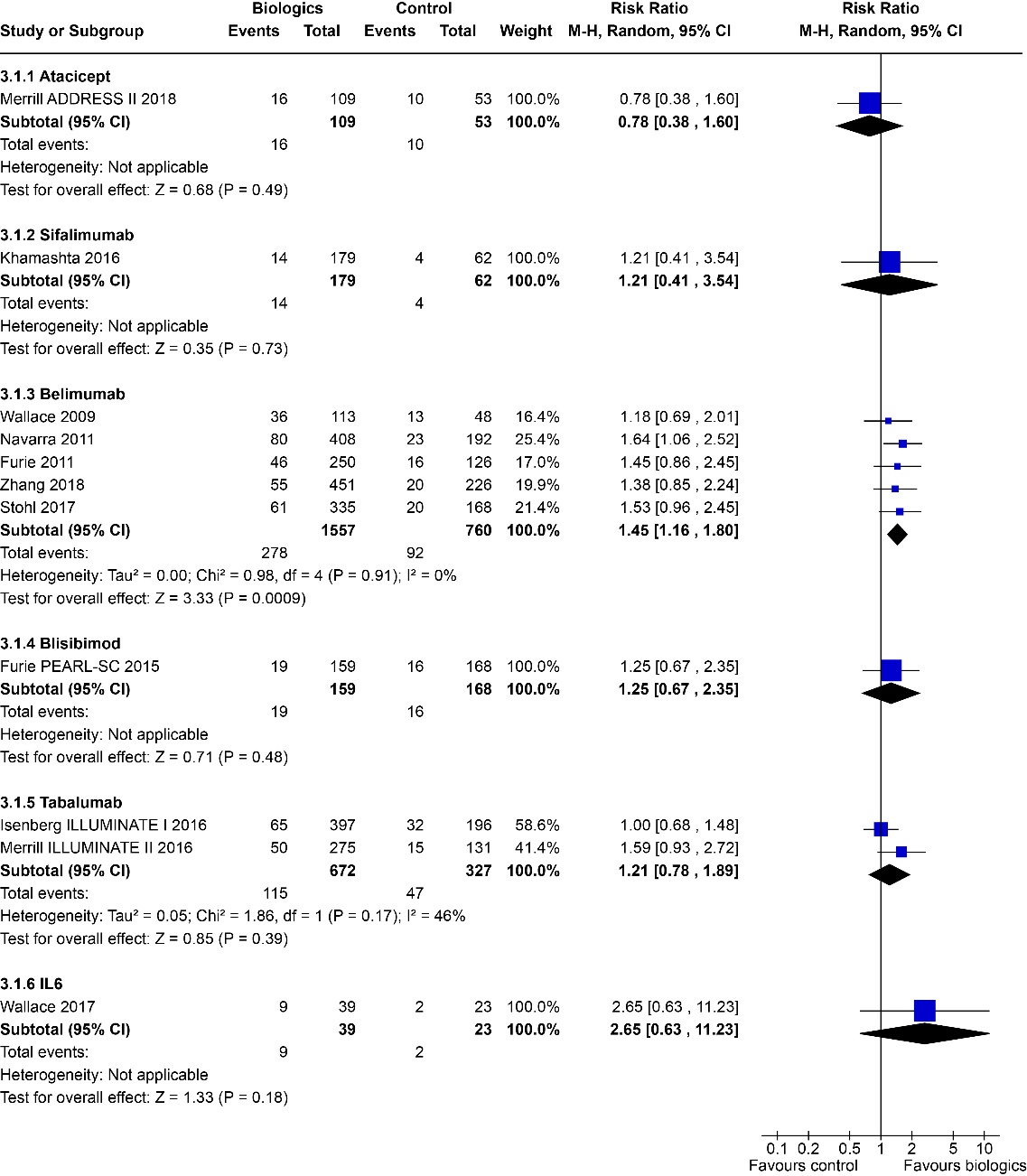


Figure 16 Change in prednisone dosages to ≤7.5mg and >25% reduction from baseline dosage


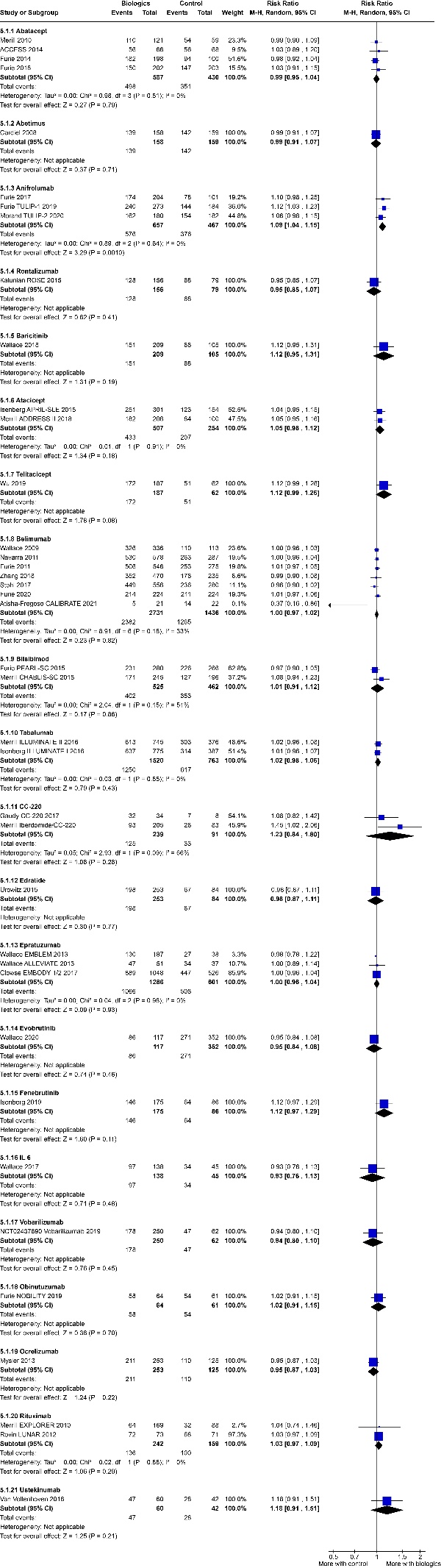


Figure 17 Adverse events


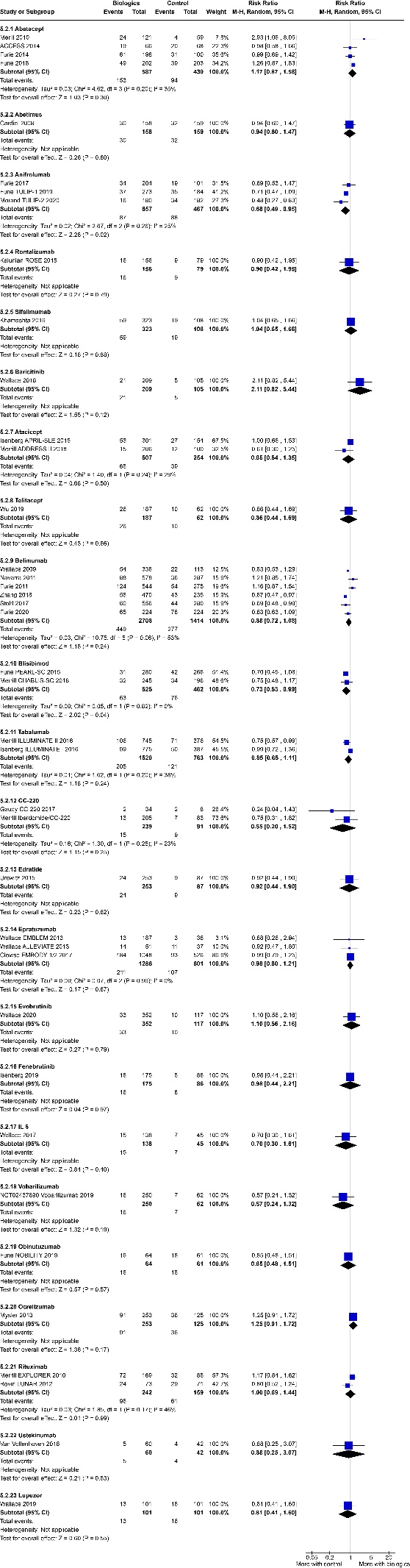


Figure 18 Serious adverse events


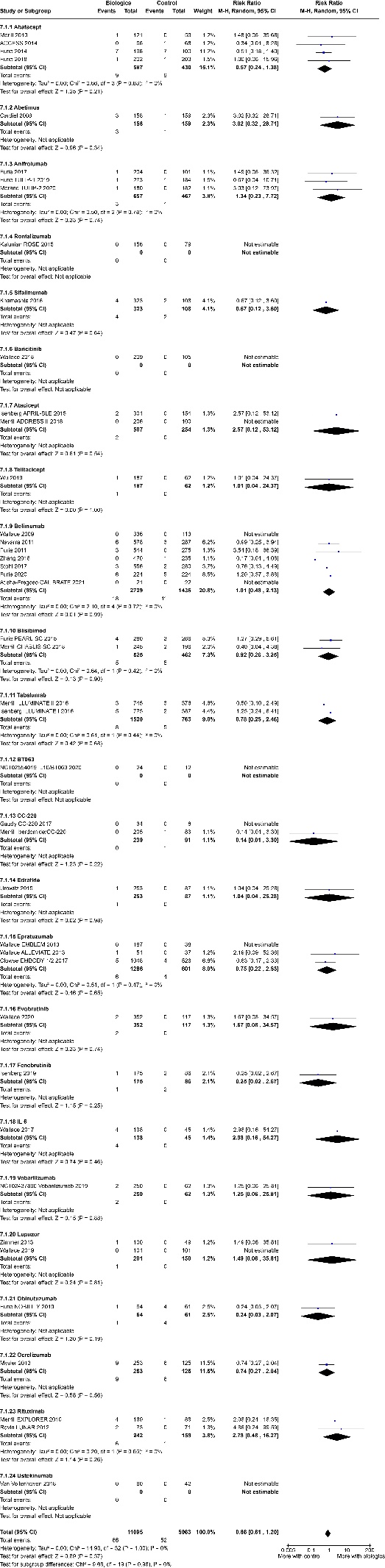


Figure 19 Death


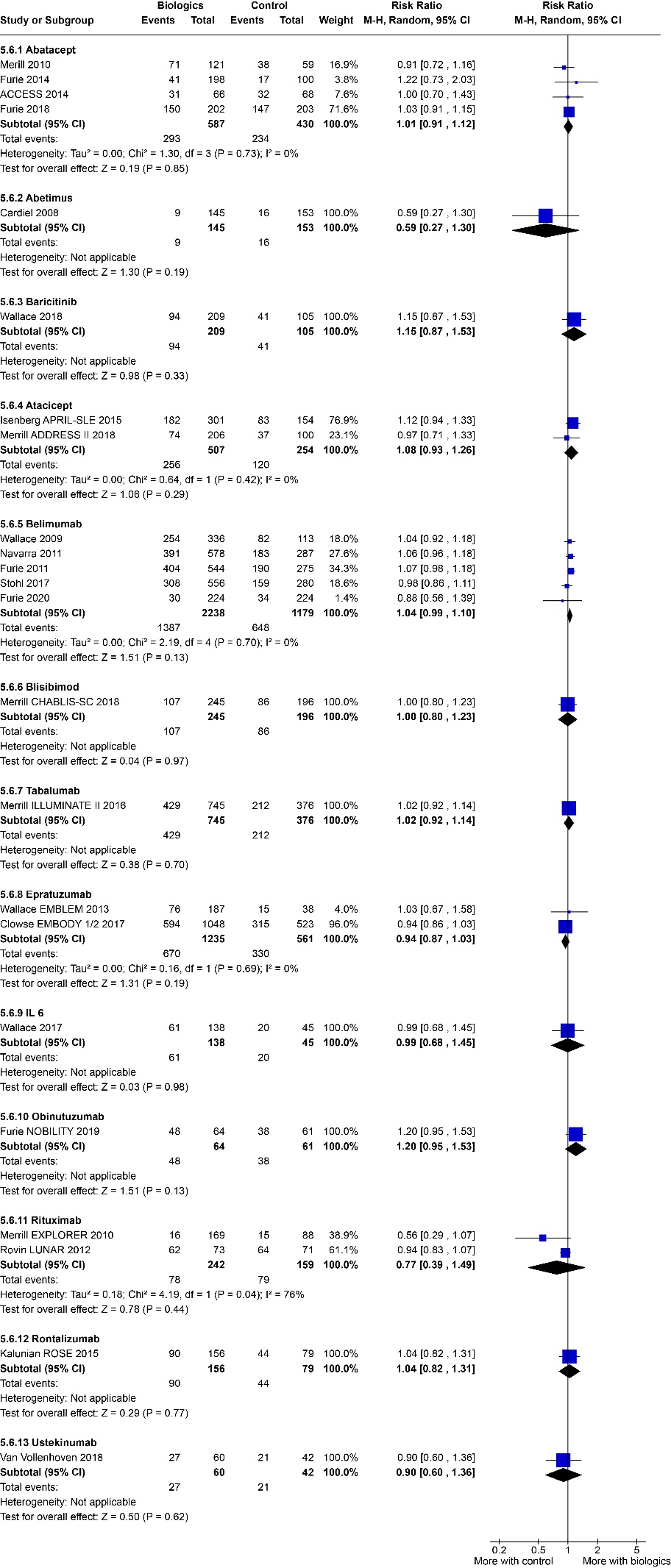


Figure 20 Infectious adverse events


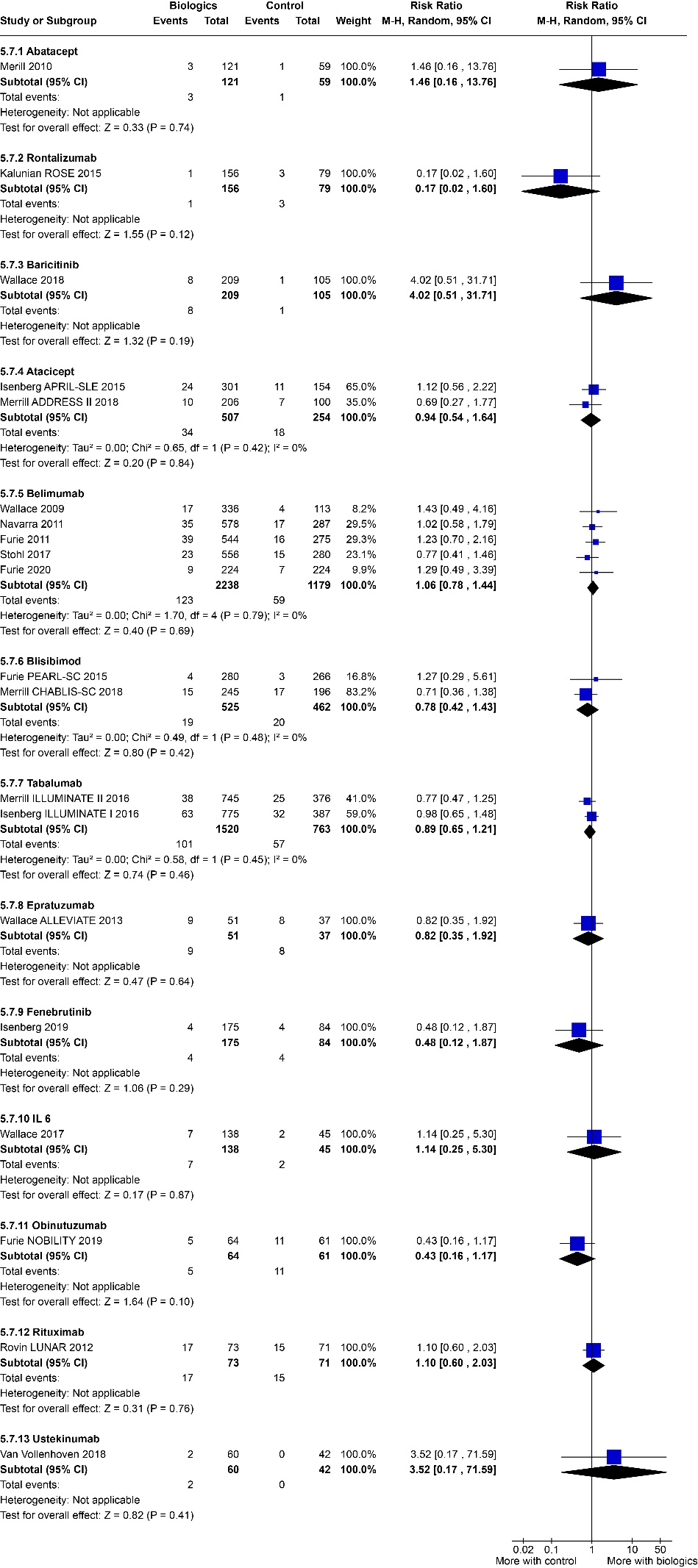


Figure 21 Serious infectious adverse events


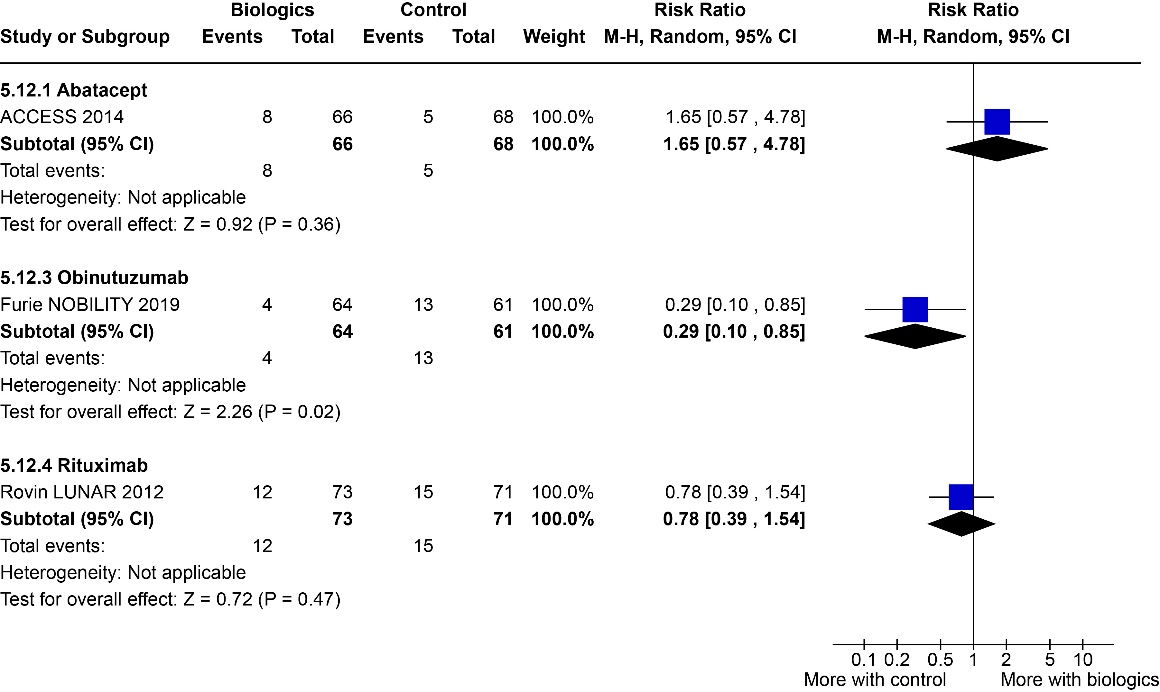


Figure 22 Grade 3 or higher infectious events


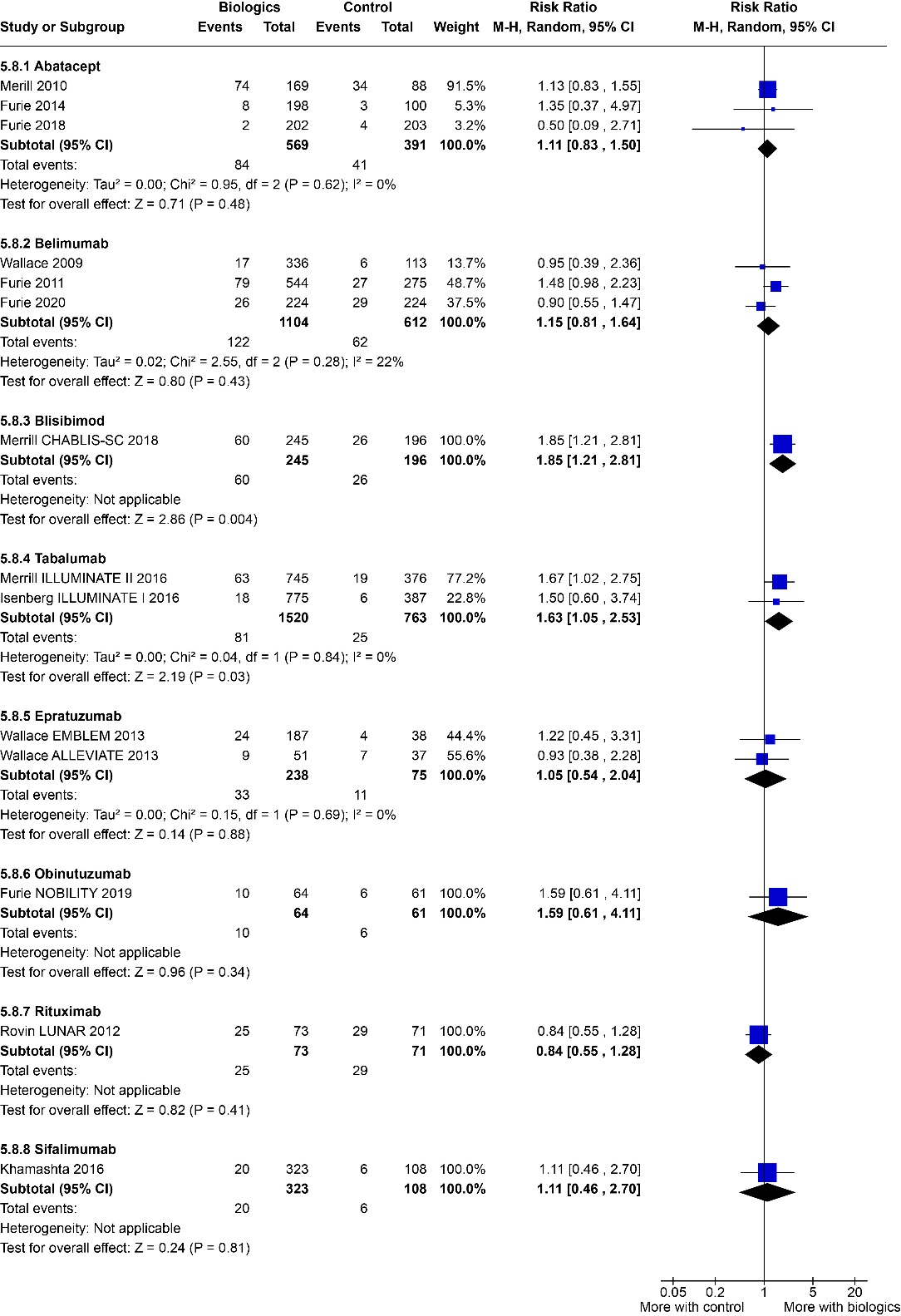


Figure 23 Infusion related adverse events


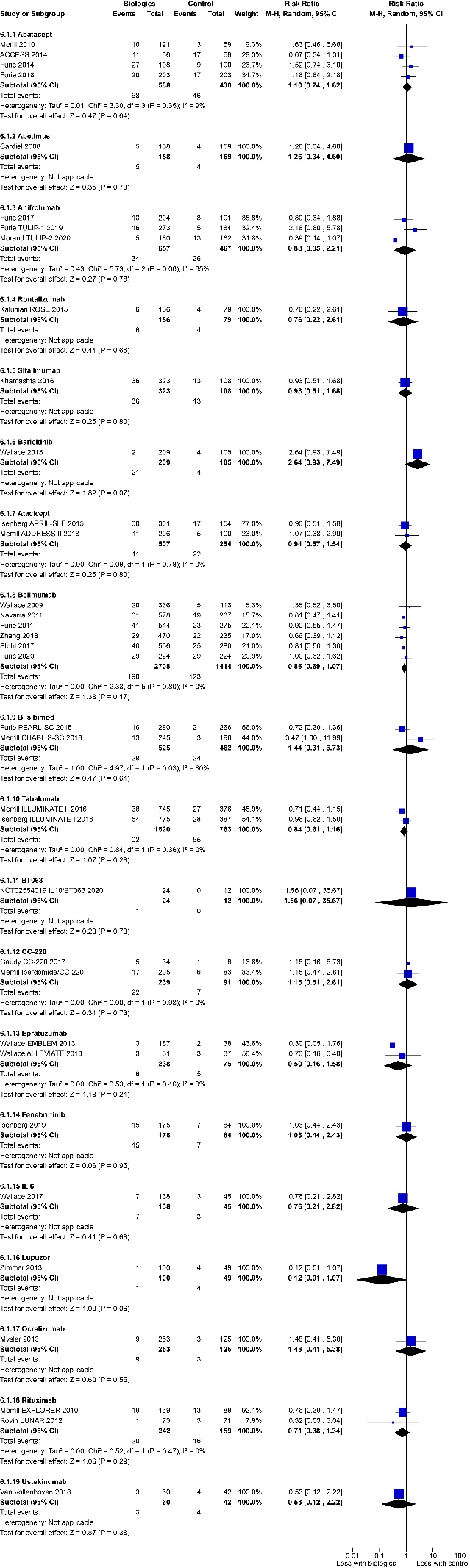


Figure 24 Withdrawal due to adverse events


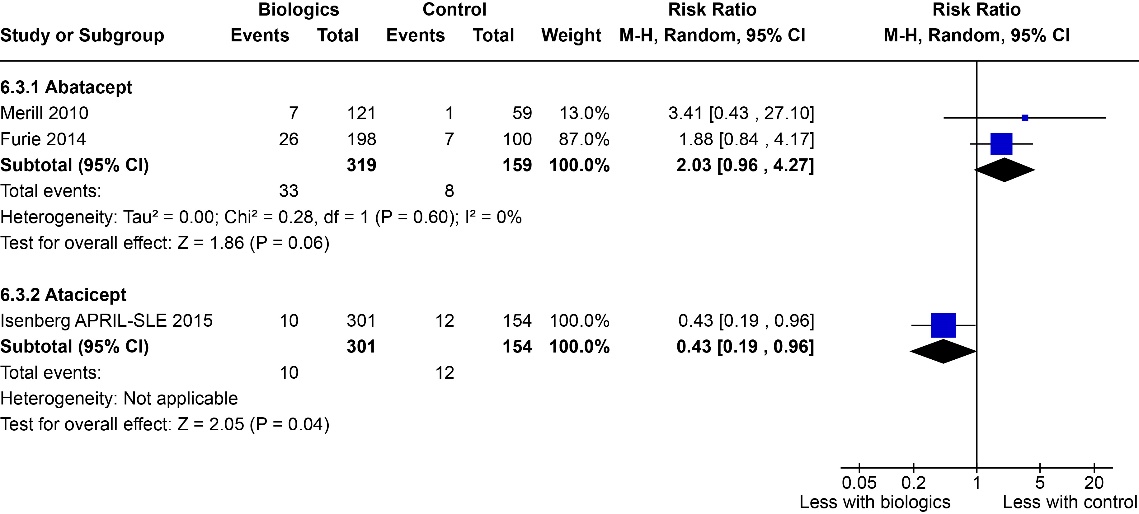


Figure 25 Withdrawal due to serious adverse events


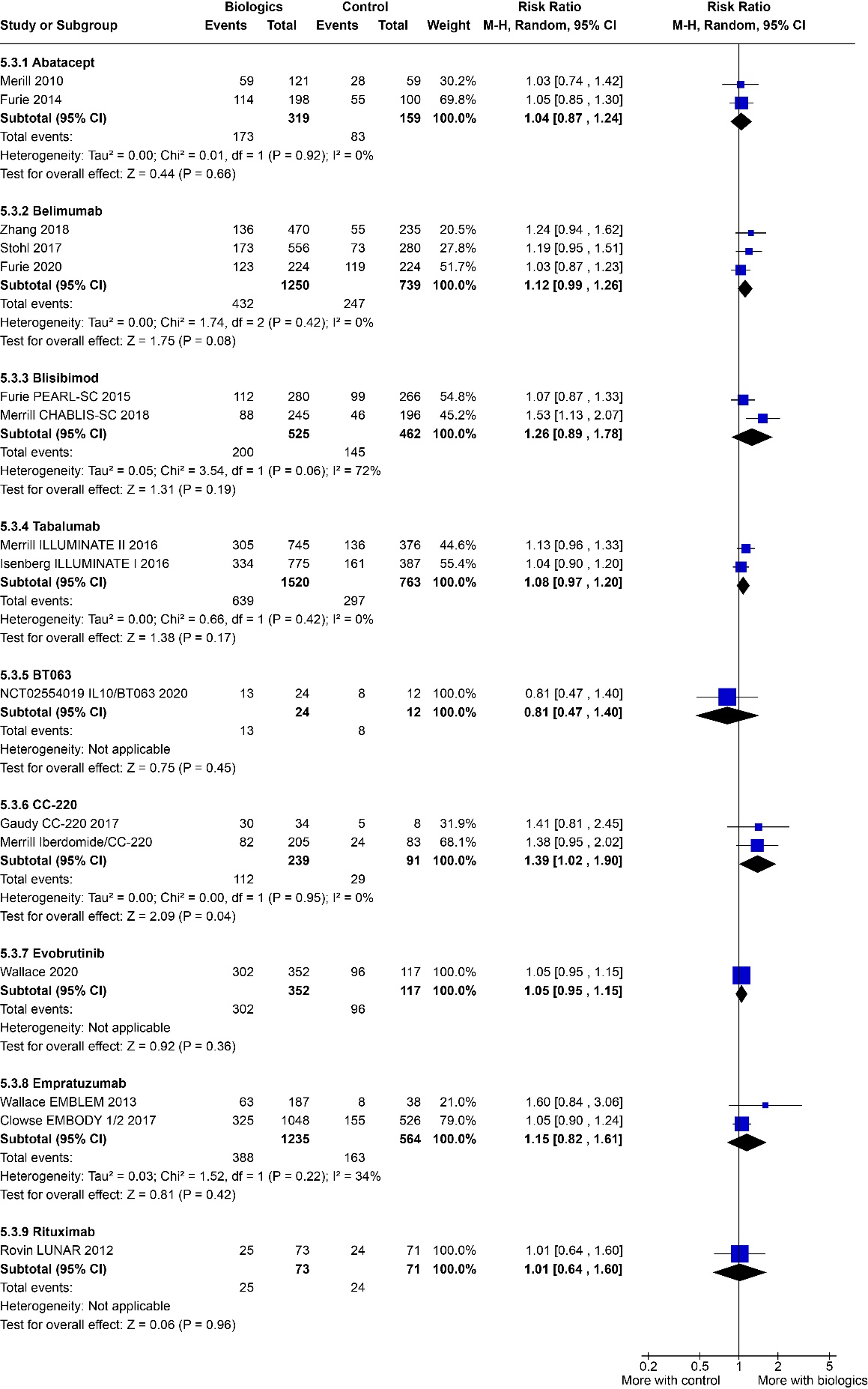


Figure 26 Treatment related adverse events


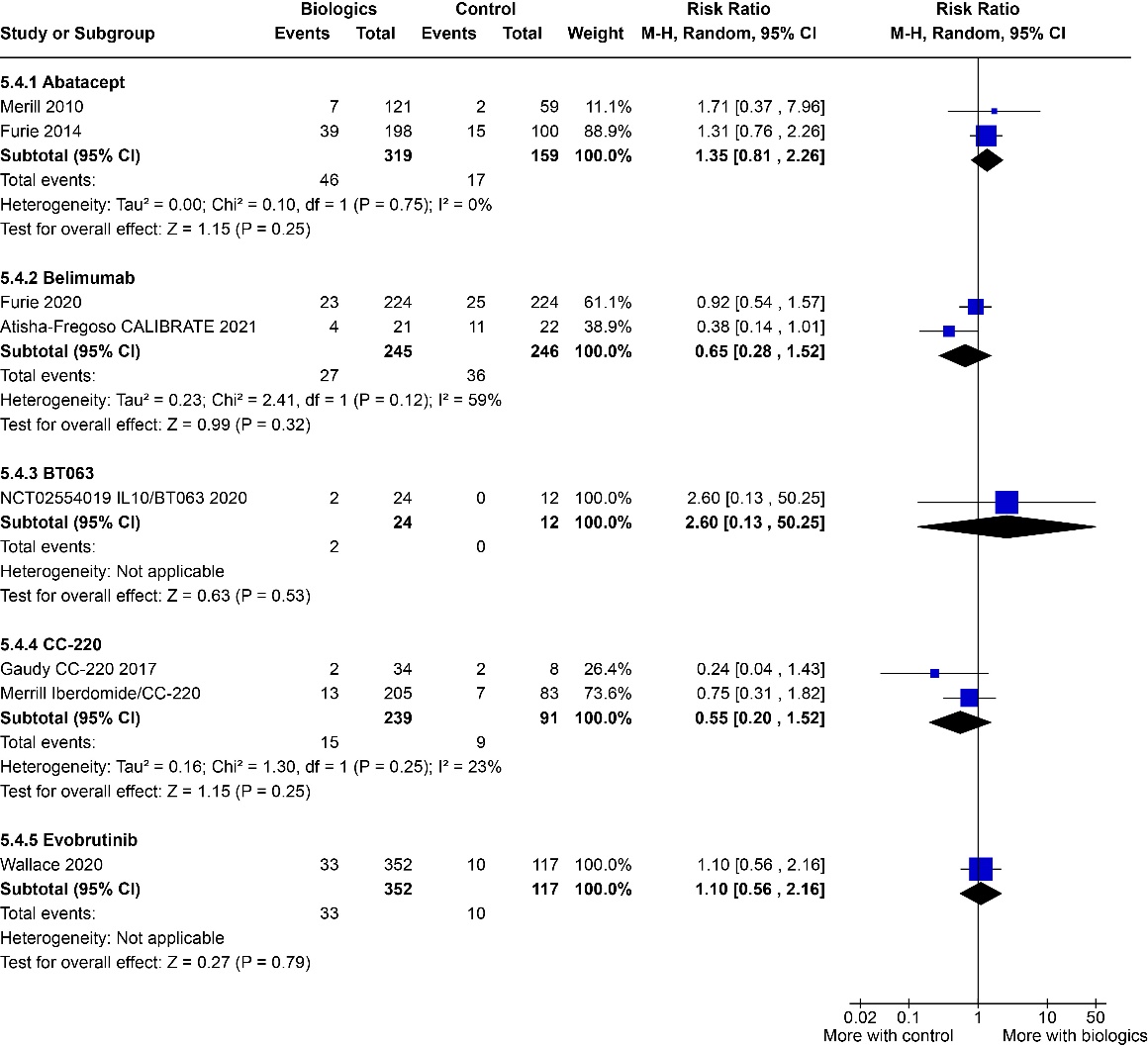


Figure 27 Serious treatment related adverse events
